# *Polygonum barbatum* extract reduces colorectal cancer cell proliferation, migration, invasion, and epithelial–mesenchymal transition via YAP and β-catenin pathway regulation

**DOI:** 10.1038/s41598-023-45630-1

**Published:** 2023-10-26

**Authors:** Pi-Kai Chang, I-Chuan Yen, Wei-Cheng Tsai, Shih-Yu Lee

**Affiliations:** 1 Graduate Institute of Medical Sciences, National Defense Medical Center, Taipei, Taiwan; 2 Division of Colon and Rectal Surgery, Department of Surgery, Tri-Service General Hospital, National Defense Medical Center, Taipei, Taiwan; 3 School of Medicine, National Defense Medical Center, Taipei, Taiwan; 4 School of Pharmacy, National Defense Medical Center, Taipei, Taiwan; 5 Graduate Institute of Aerospace and Undersea Medicine, National Defense Medical Center, Taipei, Taiwan

**Keywords:** Cancer, Molecular biology, Plant sciences

## Abstract

Colorectal cancer (CRC) is the third most common cancer worldwide with novel therapeutic developmental challenges. *Polygonum barbatum* has anticancer potential, but its mechanism(s) are unclear. This study investigates the inhibitory effect of *P. barbatum* on human CRC cells. *Polygonum barbatum* extract (PBE) and quercetin standard HPLC fingerprints were determined using analytical RP-HPLC and evaluations were completed using the human colon cancer cell line HCT-116 (KRAS^G13D^ mutation) and HT-29 (BRAF mutation) cells. Post-PBE treatment, cell viability, colony formation, migration, invasion, and apoptosis, as well as changes in the whole-transcriptome of cells were analyzed. PBE significantly reduced CRC cell growth, migration, and invasion, and the genes responsible for extracellular matrix (ECM) organization, cell motility, and cell growth were suppressed by PBE. The differentially expressed genes revealed that PBE treatment exerted a significant effect on the ECM interaction and focal adhesion pathways. Epithelial-to-mesenchymal transition markers, N-cadherin, vimentin, SLUG, and SNAIL, were shown to be regulated by PBE. These effects were associated with blockade of the Yes-associated protein and the GSK3β/β-catenin axis. PBE exerts a significant inhibitory effect on CRC cells and may be applicable in clinical trials.

## Introduction

Colorectal cancer (CRC) is the third most commonly diagnosed cancer in the world, and both its incidence and mortality rates are increasing in Asia^1^. Treatments for unresectable metastatic CRC are designed to facilitate tumor shrinkage and control metastatic lesions, and a combination of targeted therapy and cytotoxic chemotherapies is commonly applied as the primary treatment for metastatic CRC. These treatments have resulted in a significant improvement in the median overall survival, from 12 to 30 months, over the last 2 decades^2^. Most patients experience some initial response to treatment, but many experience a degree of drug resistance over time, reducing efficacy. Additionally, the high degree of toxicity associated with the chemotherapy options for CRC also limits their long-term application. Therefore, there is still an urgent need to develop novel therapeutic agents for CRC.

The Hippo pathway, including core kinase complexes MST1/2 and LATS1/2 and downstream effectors Yes-associated protein (YAP) and transcriptional coactivator with PDZ-binding motif (TAZ), regulates cell growth and differentiation acting as a tumor suppressor pathway^3^. Once activated, the Hippo pathway suppresses the nuclear translocation of YAP, which acts as an oncogene in CRC^4–6^. The Hippo pathway is downregulated in a variety of cancer cells where YAP is known to be activated. YAP promotes the expression of various target genes, including connective tissue growth factor (CTGF) and cysteine-rich angiogenic inducer 61 (CYR61), which are associated with mesenchymal differentiation^7^ and poor prognosis in CRC patients^8^. Additionally, the WNT signaling pathway regulates cell growth, epithelial-mesenchymal transition (EMT), and self-renewal, and aberrant WNT signaling has been associated with progression in CRC tissues^9, 10^. Therefore, these pathways are ideal targets for new CRC therapies.

*Polygonum barbatum,* a perennial herb belonging to *the Polygonaceae* family, is widely distributed across Southeast Asia and generally grows in marshy ground near riversides and other aquatic environments^11^. *Polygonum barbatum* is known to possess antimicrobial activity^12^ and its bioactive compounds have demonstrated anti-proliferative activity against non-small cell lung carcinoma (NCI-H640), breast cancer (MCF-7), cervical cancer (HeLa) cells^13^, and no brine shrimp toxicity^14^. However, the effect of *P. barbatum* treatment on CRC cells has not been described, and mechanistic insights into its action remain scarce. This study was designed to clarify the effects and underlying mechanism of *P. barbatum* extracts (PBE) on CRC cells.

## Results

### HPLC analysis of PBE

**Figure 1:**
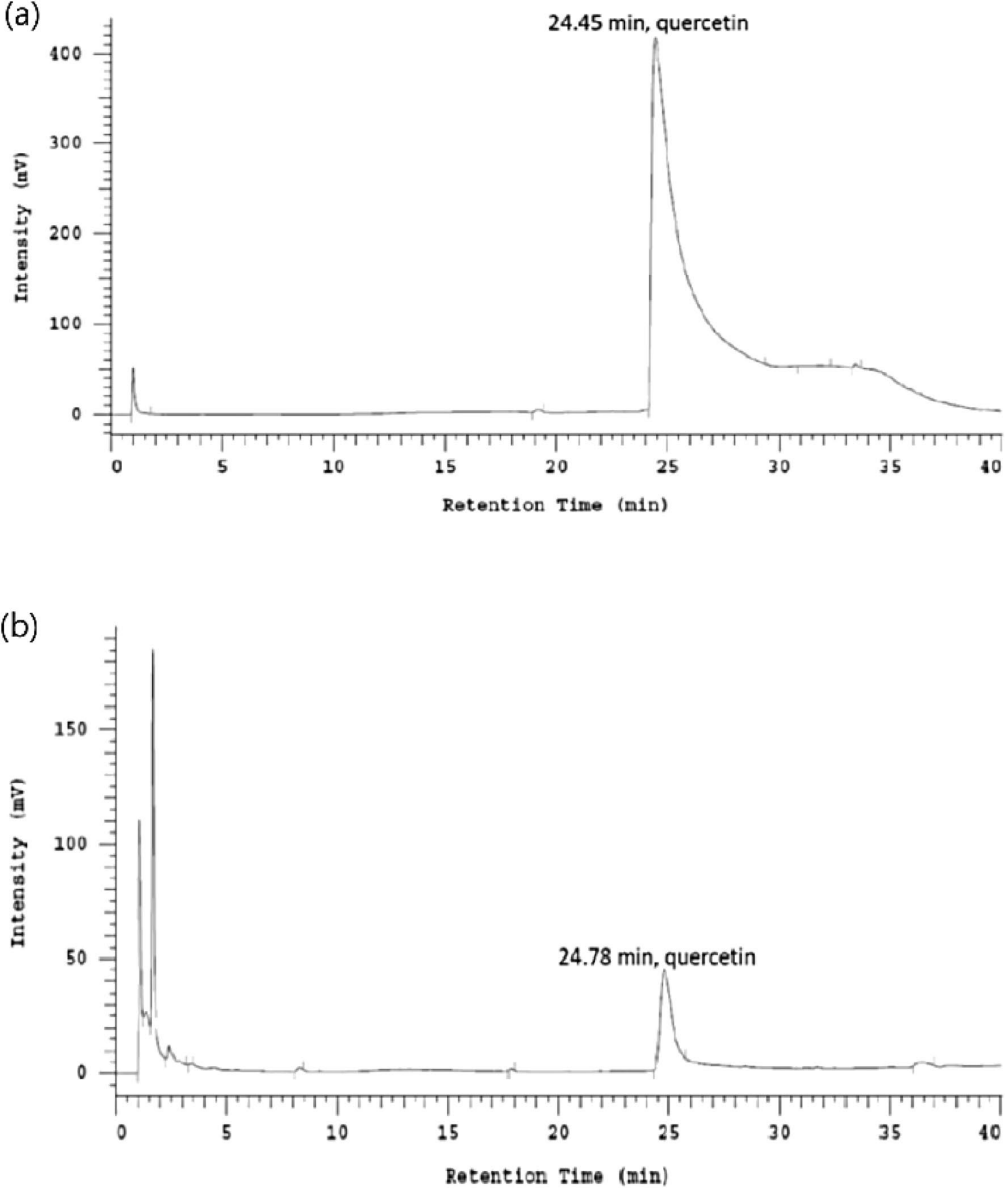
HPLC fingerprint of the quercetin (**a**) and *Polygonum barbatum* extract (PBE) (**b**). The amount of the active marker (quercetin) was determined using analytical RP-HPLC and a methanol–water gradient. The peaks were detected by UV light, and the analytes were quantified at 254 nm.

The retention time of quercetin was 24.45 min (Fig. 1a) and we produced a clear HPLC fingerprint for PBE (Fig. 1b).

### PBE significantly inhibits cell growth and induces apoptosis in CRC cells

**Figure 2:**
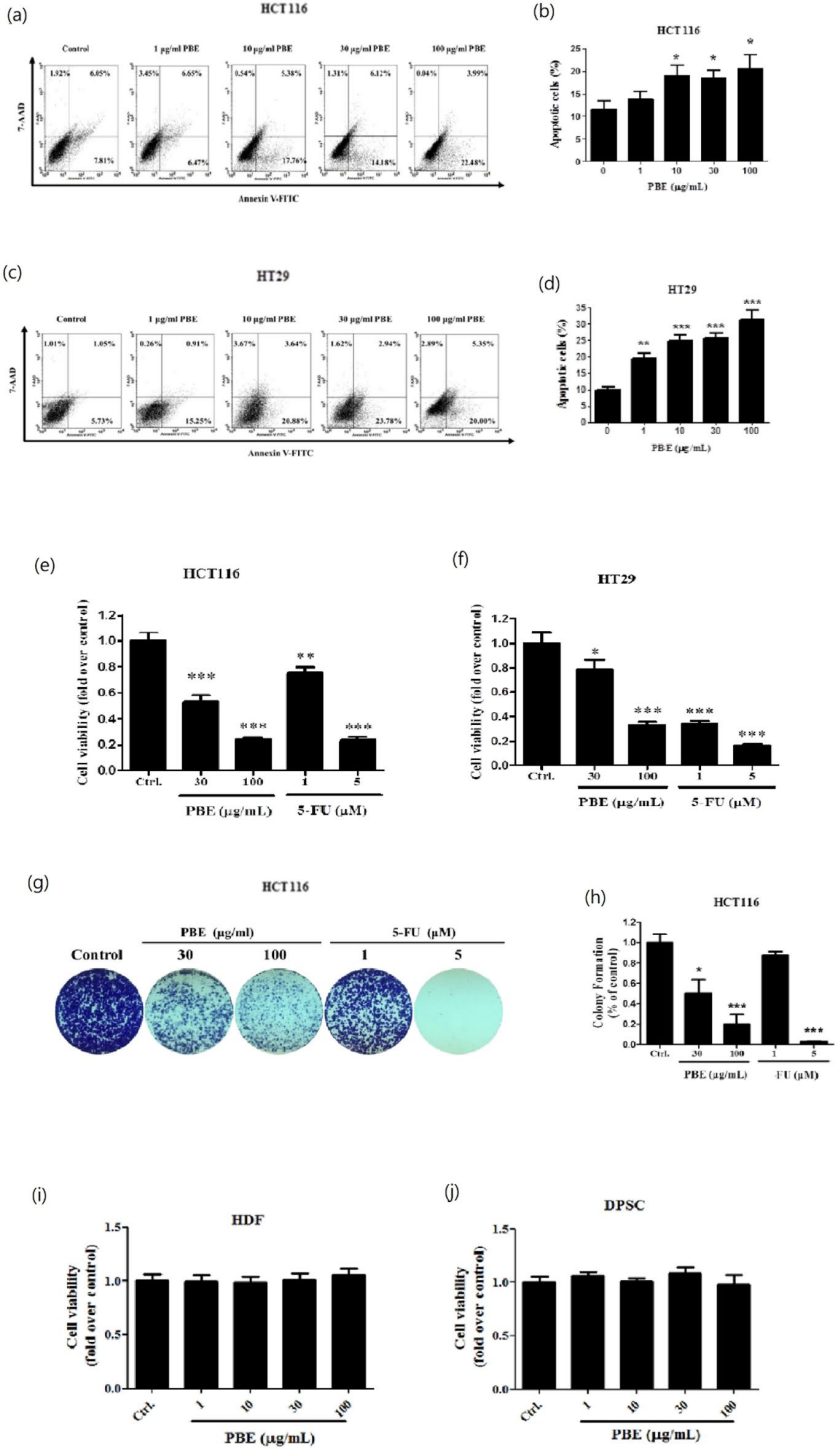
Effects of PBE treatment on apoptosis and viability in CRC cell lines. HCT116 and HT29 cells were treated with different concentrations (1–100 μg/mL) of PBE and then evaluated for apoptosis using annexin V/7-AAD double staining at 72 h (**a**,**c**). Percentage of annexin V-positive cells (**b**,**d**). Cell viability was measured using a CCK-8 Kit following 72 h of PBE exposure, 5-fluorouracil (5-FU) was used as a positive control (**e**,**f**). Colony formation was visualized using crystal violet staining following 14 days of PBE treatment (1X magnification) (**g**). Quantitative analysis of the colony formation assay, 5-fluorouracil (5-FU) was used as a positive control (**h**). Cell viability in non-cancer cells—the HDFs and DPSCs were measured using a CCK-8 Kit following 72 h of PBE exposure (i and j). Data are presented as the mean ± SD from three independent experiments. *p < 0.05, **p < 0.01, and ***p < 0.001 versus vehicle-treated cells.

Annexin V/7-AAD staining showed that PBE treatment significantly increased the number of apoptotic cells in both HCT116 and HT29 cells (Fig. 2a–d). These results were further confirmed by both cell viability (Fig. 2e–h) as non-cancer cells and colony formation (Fig. 2i–j and Supplemet Fig. 1a,b) assays with both showing significant reductions in response to PBE treatment. 5-Fluorouracil (5-FU) was used as the positive control. Human dermal fibroblast (HDF) and dental pulp stem cell (DPSC) are non-cancer cells. Combined, these results clearly indicate that PBE significantly reduces cell growth and induces apoptosis in CRC cells.

### Differentially expressed mRNAs regulated by PBE

**Figure 3:**
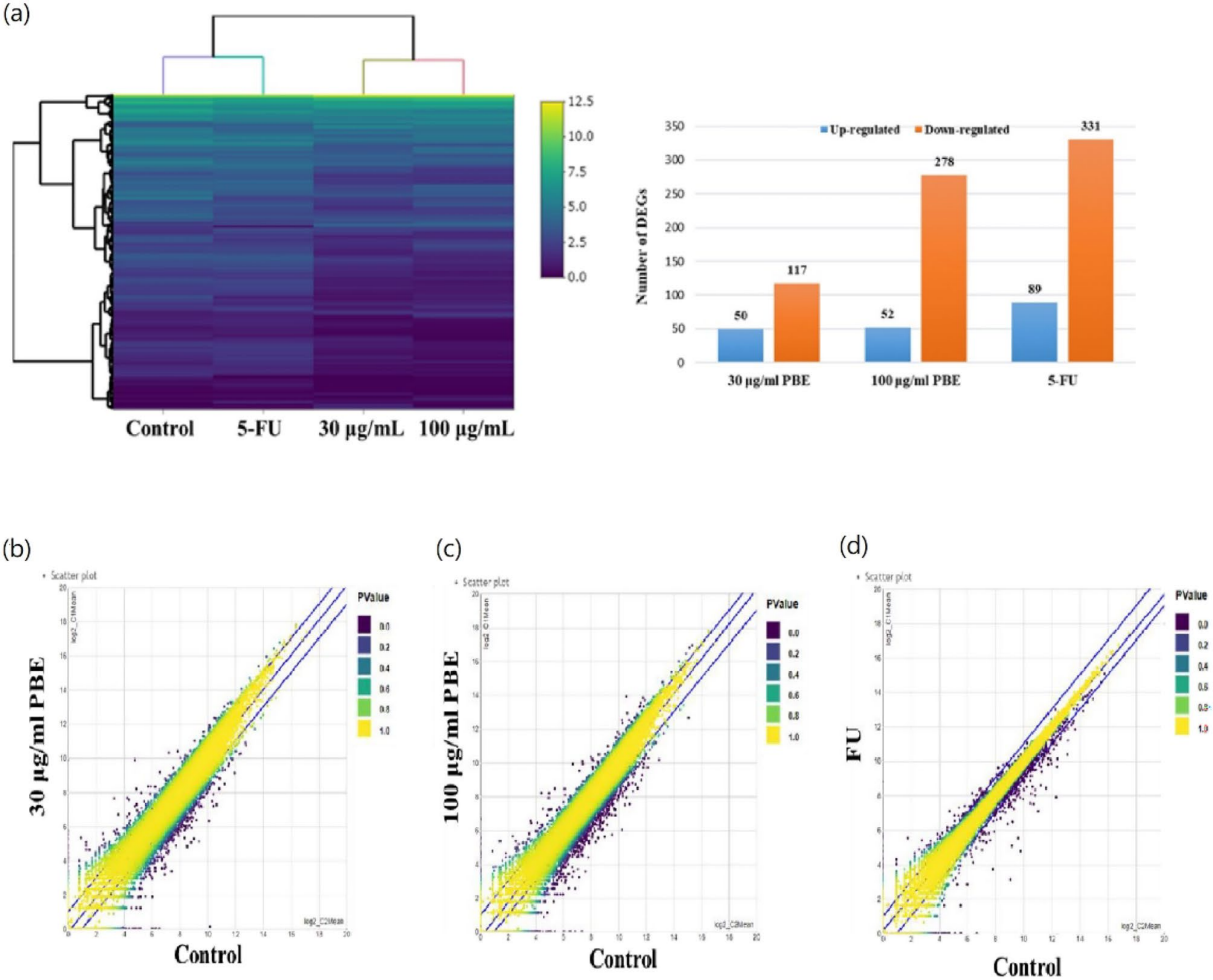
Transcriptomic profiles of PBE-treated HCT116 cells were determined using next-generation sequencing (NGS). HCT116 cells were treated with vehicle, PBE (30 and 100 μg/mL), or FU (1 μM) and then subjected to RNA sequencing. Heatmap of fragments per kilobase per million and number (FPKM) of DEGs (**a**) and DEG scatterplots (**b**–**d**) are shown. Scatterplots illustrate the correlation between gene abundance, with each data point representing gene expression levels observed in two distinct experiments. The x and y axes represent gene expression levels transformed into a log2 scale. Precisely, the x-axis corresponds to gene expression values in the control group, while the y-axis corresponds to values in the treatment group. Significant differentially expressed genes are represented as purple dots, while yellow dots indicate no significant difference. Purple dots located above the blue line signify up-regulated genes, whereas those below the blue line represent down-regulated genes.

RNA sequencing of PBE-treated HCT-116 cells was used to identify the potential mechanism of action for PBE in CRC. The quality and read data for this sequencing experiment are summarized in Supplement Table 1. The Q30 statistics of the clean reads in all samples exceeded 95%, indicating high quality samples. We identified 167 and 330 differentially expressed genes (DEGs) in cells treated with 30 μg/mL PBE (117 downregulated and 50 upregulated) and 100 μg/mL PBE (278 downregulated and 52 upregulated), respectively (Fig. 3a). DEG scatterplots show the differences between the vehicle control and 30 μg/mL PBE (Fig. 3b), 100 μg/mL PBE (Fig. 3c), or 5-FU (Fig. 3d), respectively.

### Kyoto encyclopedia of genes and genomes (KEGG) pathway and gene ontology (GO) enrichment analysis

**Figure 4:**
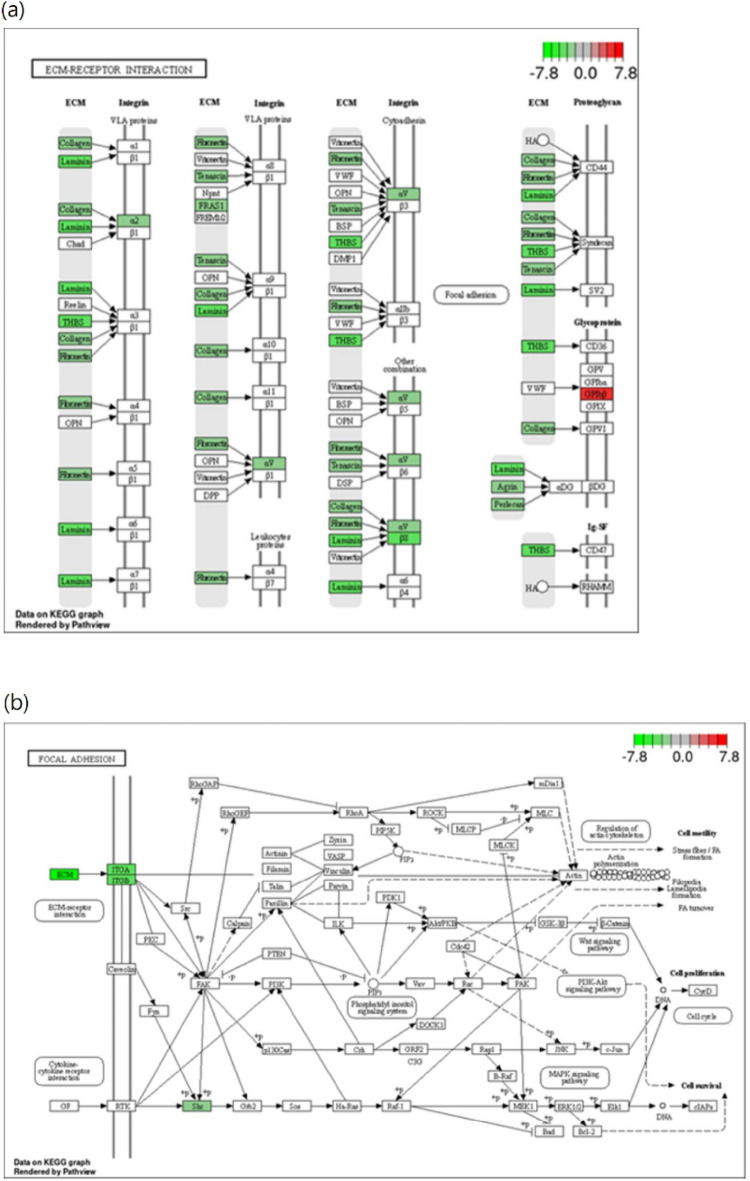
KEGG Pathway Enrichment Analysis. ECM-receptor interactions (**a**) and FA (**b**) signaling pathways were downregulated in response to PBE when evaluated by KEGG.

KEGG ( https://www.genome.jp/kegg/) and GO ( https://geneontology.org/) analysis were then used to further clarify the regulatory signaling pathway involved in PBE mediated inhibition of CRC cell proliferation, and the top 10 KEGG pathways for the DEGs in each group are shown in Supplemet Tables 2–4. We found that extracellular matrix (ECM)-receptor interactions and focal adhesion (FA) were the most significantly enriched pathways in PBE-treated cells (Fig. 4a,b) and that these pathways included thrombospondin 1 (THBS1), glycoprotein Ib platelet subunit beta (GP1BB), laminin subunit alpha 5 (LAMA5), AGRN, integrin subunit beta 8 (ITGB8), tenascin XB (TNXB), heparan sulfate proteoglycan 2 (HSPG2), Fraser ECM complex subunit 1 (FRAS1), integrin subunit alpha 2 (ITGA2), fibronectin 1 (FN1), laminin subunit beta 2 (LAMB2), collagen type IV alpha 5 (COL4A5), integrin subunit alpha V (ITGAV), and SHC adaptor protein 3 (SHC3). Additionally GO enrichment analysis of the DEGs from the PBE-treated groups displayed significant enrichment for cellular proliferation and ECM organization (Supplemet Fig. 2a,b). Whole blood vessel morphogenesis was shown to be significantly enriched in the FU group (Supplemet Fig. 2c). When we evaluated the GO terms associated with molecular function we found that both integrin binding and extracellular binding were significantly enriched in the PBE-treated cells (Supplemet Fig. 2d,e), while RNA polymerase II core promoter proximal region sequence-specific DNA binding was enriched in the 5-FU-treated cells (Supplemet Fig. 2f). Furthermore, both endocytic vehicle lumen and extracellular cellular components from the cellular component category were significantly enriched in the PBE-treated cells (Supplemet Fig. 2g,h), while adherens junctions was the most significantly enriched term in the FU-treated cells (Supplemet Fig. 2i).

### PBE significantly inhibits cell migration, invasion, and epithelial-to-mesenchymal transition (EMT) in CRC cells

**Figure 5:**
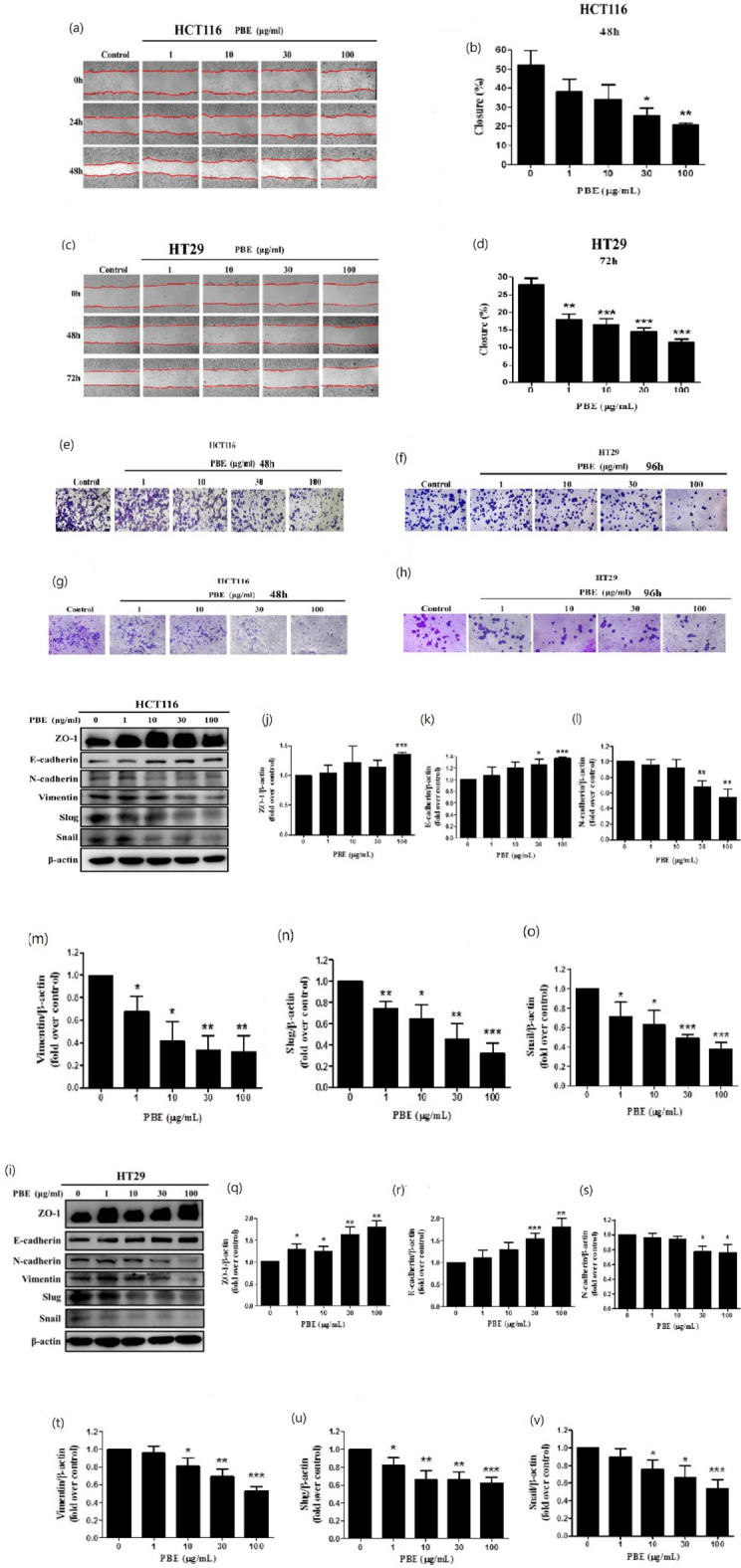
Effect of PBE on cell migration, invasion, and EMT. Cell migration was analyzed using both scratch (40X magnification in **a** and **c**) (**a**–**d**) and migration assays (100X magnification) (**e**,**f**) following 48 h or 72 h of treatment with PBE. Cellular invasion assays were completed following 48 h or 96 h of treatment with PBE (100X magnification) (**g**,**h**). EMT markers in both HCT116 (**i**–**o**) and HT29 (**p**–**v**) cells were evaluated by western blot following 72 h of treatment with PBE. Data are presented as the mean ± SD from three independent experiments. *p < 0.05, **p < 0.01, and ***p < 0.001 versus vehicle-treated cells.

ECM-receptor interactions and FA are highly associated with cell migration, invasion, and EMT in CRC cells. To confirm, wound healing, cell migration, and invasion assays were used to elucidate the effects of PBE on CRC cells. We found that PBE significantly inhibited the wound healing rate in both HCT-116 (Fig. 5a,b) and HT-29 (Fig. 5c,d) cells. Similar inhibitory effects were observed in the migration assays (Fig. 5e,f) and we found that the invasion rates of PBE-treated cells were significantly lower than those of the vehicle control (Fig. 5g,h). We went on to evaluate the EMT-associated markers using western blotting. We found that the levels of epithelial markers ZO-1 and E-cadherin were increased in PBE-treated cells when compared with the control in both HCT-116 (Fig. 5i–k) and HT-29 cells (Fig. 5p–r). Contrastingly, mesenchymal markers, including N-cadherin, vimentin, SLUG, and SNAIL all decreased in response to PBE treatment in both HCT-116 (Fig. 5i,l,m,n,o) and HT-29 cells (Fig. 5p,s–v). These findings indicate that PBE inhibits migration, invasion, and EMT in CRC cells.

### PBE blocks the YAP signaling pathway

**Figure 6:**
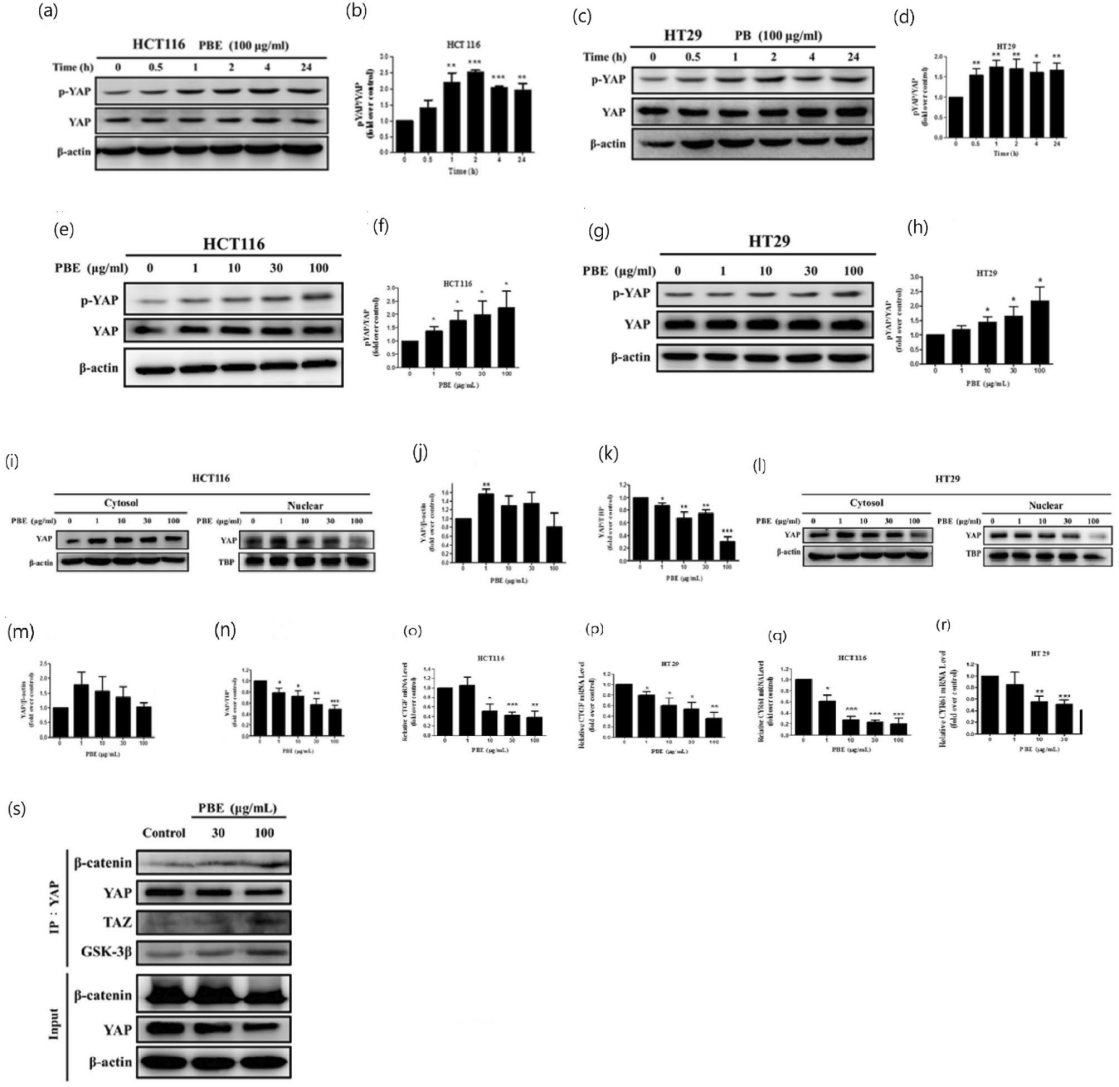
Effect of PBE on the YAP signaling pathway. Phosphorylation of YAP following PBE treatment was analyzed using western blotting and evaluated in terms of both time (**a**–**d**) and dose dependency (**e**–**h**). Nuclear and cytosolic translocation of YAP was also evaluated in both cell lines (**i**–**n**). The relative expression of CTGF and CYR61 was determined using quantitative PCR (**o**–**r**). Co-IP was used to evaluate the interactions between YAP, β-catenin, and GSK3β in HCT116 cells (**s**). Data are presented as the mean ± SD from three independent experiments. *p < 0.05, **p < 0.01, and ***p < 0.001 versus vehicle-treated cells.

To further explore the molecular mechanisms underlying the effects of PBE in CRC cells, western blot was used. We found that 100 μg/mL PBE significantly increased the S127 phosphorylation of YAP in both cell lines (Fig. 6a–d). Moreover, the levels of p-YAP increased in a dose-dependent manner following the addition of PBE (Fig. 6e–h). This was then confirmed by evaluating both cytoplasmic and nuclear extracts in more detail. PBE treatment significantly reduced the nuclear translocation of YAP, but did not change the cytosolic retention of this protein (Fig. 6i–n). Meanwhile, PBE treatment reduced the expression of YAP target genes CTGF and CYR61 (Fig. 6o–6r). These results reveal that PBE treatment significantly suppresses YAP signaling. Additionally, co-immunoprecipitation implies that PBE regulates the WNT signaling pathway (Fig. 6s).

### PBE suppresses the GSK3β/β-catenin signaling pathway

**Figure 7:**
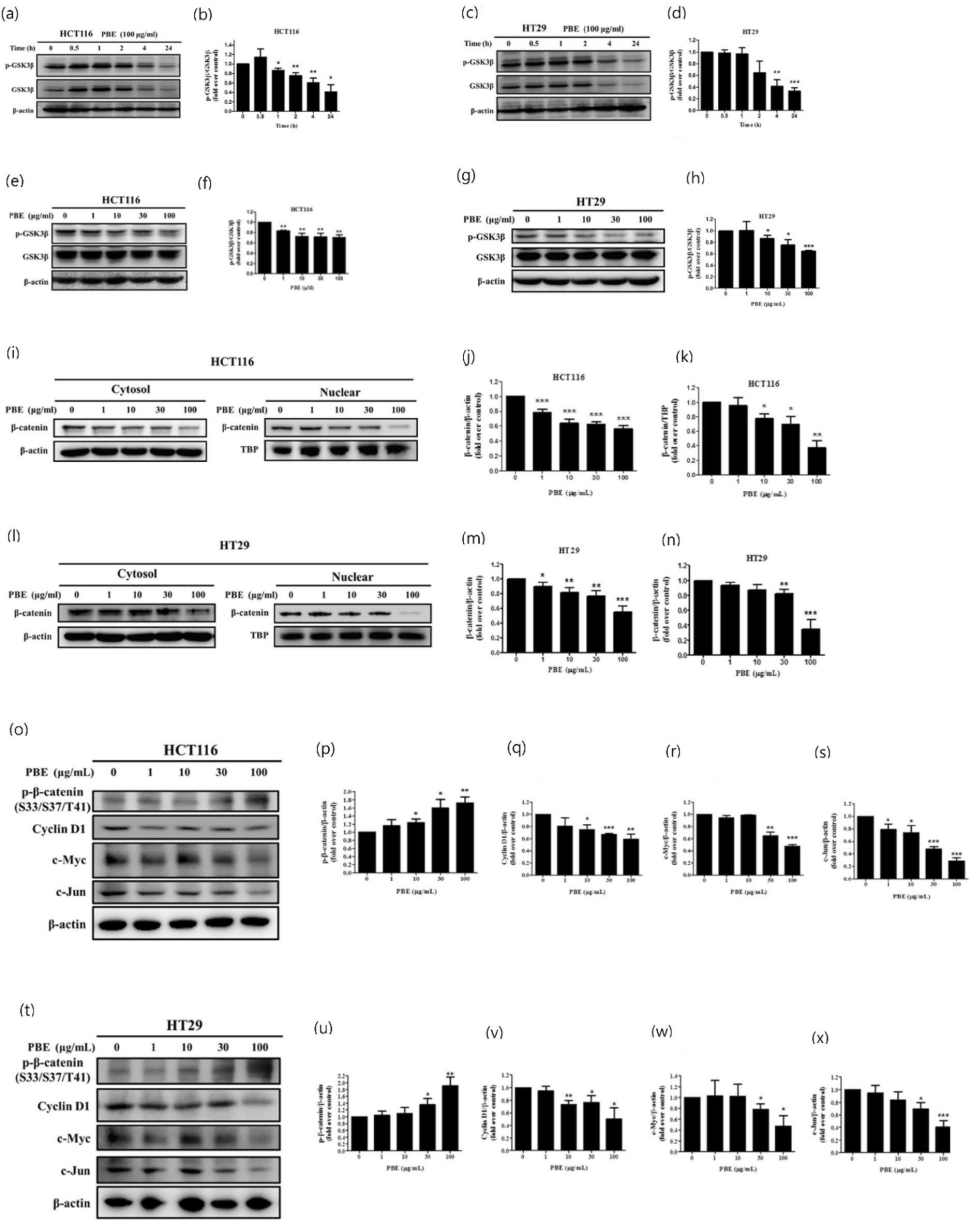
Effect of PBE on the GSK3β/β-catenin signaling pathway. Phosphorylation of GSK3β was evaluated in terms of both time (**a**–**d**) and dose dependency (**e**–**h**) using western blotting. Nuclear and cytosolic translocation of β-catenin was also evaluated in both cell lines (**i**–**n**) and the protein levels of cyclin D1, c-Myc, and c-Jun were analyzed and quantified (**o**–**x**). Data are presented as the mean ± SD from three independent experiments. *p < 0.05, **p < 0.01, and ***p < 0.001 versus vehicle-treated cells.

We then went on to confirm the role of PBE in the regulation of WNT signaling by evaluating the GSK3β/β-catenin signaling pathway. As expected, PBE significantly reduced the phosphorylation of GSK3β at Ser9 in both a time (Fig. 7a–d) and dose dependent manner (Fig. 7e–h) in both cell lines. Consistently, the nuclear and cytosolic protein levels of β-catenin were also shown to be modulated by PBE treatment (Fig. 7i–n). We further investigated the downstream targets of the WNT pathway and demonstrated that PBE treatment significantly increased the phosphorylation of β-catenin and its targets, including cyclin D1, c-Myc, and c-Jun, which were downregulated in both cell lines (Fig. 7o–x). These findings indicate that PBE inhibits the WNT/β-catenin signaling pathway in CRC cells.

### Discussion

**Figure 8:**
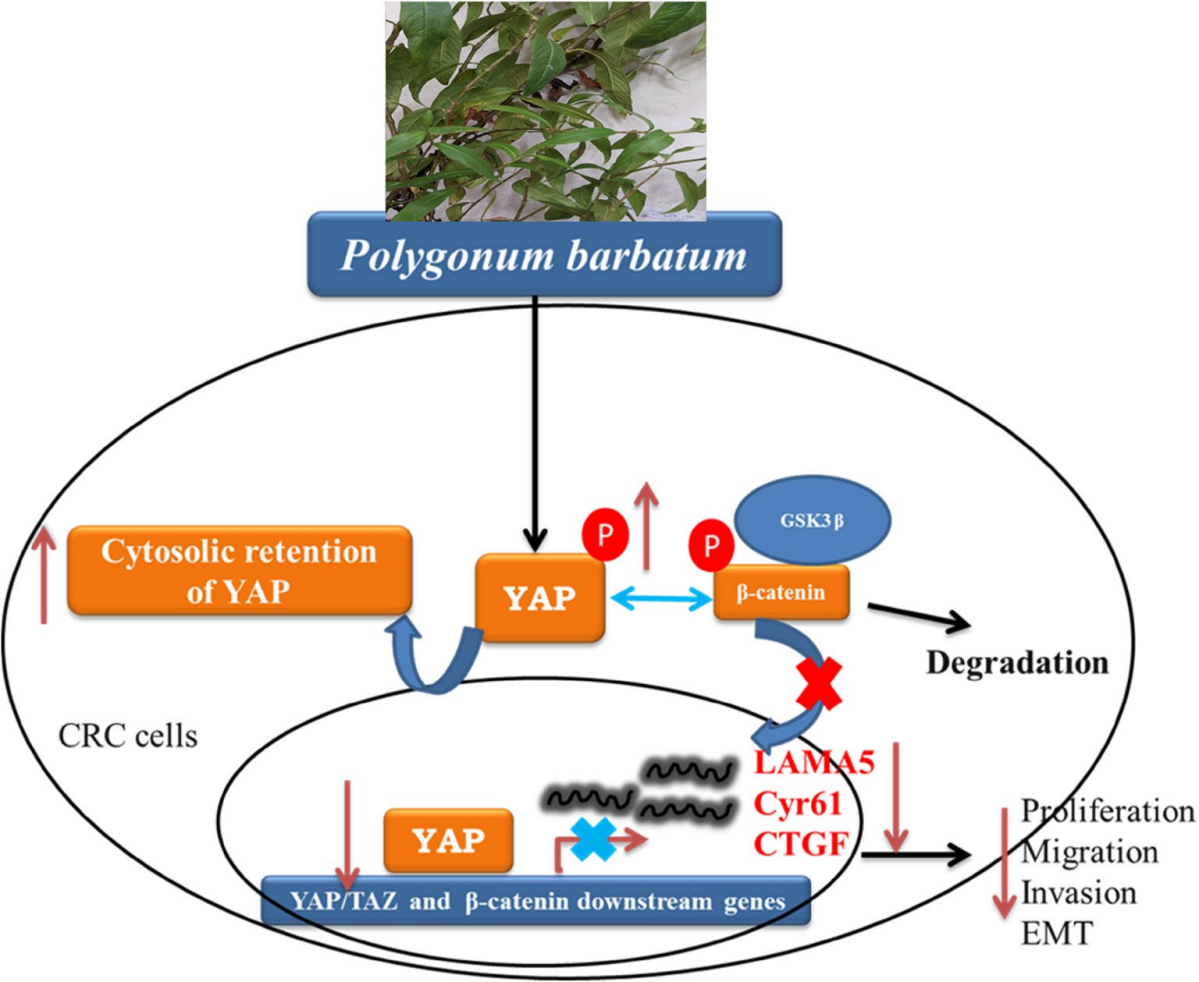
Proposed mechanisms of action for PBE in CRC cells. PBE increases the phosphorylation of YAP and blocks WNT signaling, decreasing cell adhesion and ECM stiffness, resulting in the inhibition of CRC cell invasion and migration.

PBE significantly reduced motility and tumorigenic potential by modulating EMT in both HCT116 and HT29 CRC cells. PBE treatment triggered cellular apoptosis, as established by an annexin V-FITC and 7-AAD double stain assay. Both GO and KEGG analysis of the RNA sequencing data was consistent with these findings, with this analysis demonstrating that the expression of the genes responsible for ECM organization (*CTGF* and *CYR61*), cell motility (*THBS1*), and cell growth (*FN1*) were all inhibited by PBE (Fig. 4A,B). These effects are associated with the blockage of both YAP and WNT signaling (Fig. 8).

*Polygonum barbatum* has been reported to produce potential anticancer bioactive compounds such as dihydrobenzofuran, sesquiterpene derivatives^13, 15^, and quercetin^16^. Quercetin has been shown to activate the Hippo pathway and inhibit YAP signaling^17^, and sesquiterpene derivatives have been reported to induce ROS- and TRAIL-mediated apoptosis, enhance chemotherapy responses, and inhibit EMT with these effects being accompanied by the downregulation of β-catenin in CRC cells^18^. To the best of our knowledge, this is the first study to show that* P. barbatum* can reduce the migration and tumorigenic potential of CRC cells by blocking both YAP and β-catenin signaling. Based on these findings, these bioactive compounds are thought to synergistically contribute to the anti-CRC properties of PBE. However, most of these findings are based on in vitro studies, which means that further in vivo evaluations and clinical trials are required to make any definitive statements on their activity. Interestingly, 2,3-dihydrobenzofuran derivatives have been shown to exhibit microsomal prostaglandin E2 synthase-1 inhibitor activity^19^, a key enzyme in prostaglandin E2 (PGE_2_) synthesis known to boost CRC immune evasion^20^. Therefore, the regulatory effect of PBE on the COX-2/PGE_2_ axis, and its mediators such as Janus kinase 2/signal transducer and activator of transcription 3 in CRC, will be investigated in our future work.

The ECM regulates cellular behavior and participates in both cellular adhesion and migration, with the overexpression of ITGAV, ITGA1, ITGB8, and FN genes known to be involved in CRC growth and metastasis^21–24^. Additionally collagen XII, FRAS1, LAMA5, and THBS1 are all associated with colorectal liver metastasis, which is the most common distant metastasis in CRC patients^25–27^. In this study, our GO and KEGG analyses of the RNA sequencing data revealed that both the ECM and FA pathways were significantly downregulated in response to PBE. When combined with the results from the apoptosis evaluations we can confirm that these outcomes are consistent with the finding that diminished expression of ITGA2 promotes death and apoptosis in CRC cells^28^. Interestingly, we found that GP1BB, a regulator of epithelial cell adhesion, was upregulated in response to PBE treatment. This regulator has been reported to increase cell–cell contact, leading to the downregulation of EMT^29^. Consistently, cell mobility, invasion, and EMT were also shown to be inhibited by PBE. These findings indicate that PBE is able to inhibit the ECM-receptor interaction and FA pathways, suppressing cell migration, invasion, and EMT. ECM and FA are also involved in the regulation of many signaling pathways, including the Hippo pathway. Low ECM resistance and FA correspond to reduced mechanics and subsequently regulate the Hippo pathway^30^. YAP/TAZ, the major effector of the Hippo pathway, senses alterations in ECM composition^31^ and its downstream targets CYR61 and CTGF are upregulated. This upregulation has been linked to drug resistance and RAS/MAPK blockade in CRC cells, indicating their critical role in RAS-mutated metastasis and drug resistance^32–35^. Here we found that PBE treatment blocked YAP signaling and its downstream gene expression. This indicates that PBE inhibits CRC progression by regulating YAP signaling, which is closely associated with EMT progression. PBE also inhibited several EMT markers, including SNAIL, TWIST, and SLUG. We also showed that PBE was able to inhibit cell growth, invasion, and migration in both HCT116 and HCT29 cells. Our findings were consistent with those of a recent study wherein the cell–matrix interface determined CRC dormancy, and the YAP blockade reduced cancer recurrence^36^. Based on these findings we suggest that PBE might exhibit synergistic effects with FU and EGFR inhibitors (cetuximab) when used to treat CRC. However, this requires further investigation. YAP is regulated by the Hippo pathway, including MST1/2 and LATS1/2, which interacts with the phosphatase and tensin homolog (PTEN)/AKT/mechanistic target of rapamycin (mTOR) autophagic axis^37^. However, the role of autophagy in CRC remains unclear and still requires further in-depth mechanistic studies to be fully understood^38^. Given this, it would be interesting to investigate the role of PBE in the modulation of autophagy and the regulation of YAP via the Hippo pathway.

Both of Hippo and WNT/β-catenin pathways are involved in chemoresistance and disease recurrence in CRC^4^. Furthermore, the YAP/β-catenin axis participates in proliferation, metastasis, angiogenesis and EMT in CRC^5, 39^. Consistently, the regulation of YAP/β-catenin axis shows an inhibitory effect on colorectal cancer cell growth^6^. This means that WNT/β-catenin signaling is a potential therapeutic target for CRC. Once β-catenin phosphorylated, it subsequently leads to degradation^40^. Moreover, destruction complex formation including YAP/TAZ, GSK3β, and β-catenin contributes to the WNT pathway OFF state^41^. We found that PBE treatment increased the phosphorylation of β-catenin and destruction complex formation, which blocked β-catenin-mediated oncogenic signaling and inhibited invasion, migration, and EMT progression. Hippo signaling also interacts with Notch signaling to suppress liver tumorigenesis^42^ and LAMA5 expression is associated with Notch signaling, which promotes EMT by interacting with *SLUG* and *SNAIL*^27^. Based on the inhibitory effect of PBE on EMT, it would be interesting to investigate the regulatory role of PBE in Notch signaling, which has already been tightly linked to CRC progression^43^. However, this requires further investigation. In this study, we showed that PBE exerts a significant inhibitory effect on the two main signaling pathways (YAP and Wnt/β-catenin) associated with CRC progression. However, KRAS mutations and PI3K/AKT activation play important roles in drug resistance^44^, and PBE exerts equal inhibitory effects in both KRAS mutant and BRAF mutant CRC cells. It would be interesting to investigate the synergistic effect of PBE and EGFR inhibitors in the treatment of CRC in future work.

In this study, we have conducted comprehensive experiments using various colorectal cancer cell lines, including HT29 and HCT116. However, it's important to note that the pathophysiological mechanisms of colorectal cancer are complex. Therefore, further validation through animal experiments is needed to confirm our findings. Our findings show the first time transcriptome profile targeted by *P. barbatum* and its inhibitory effect on CRC cell motility and tumorigenic potential via regulation of the YAP and β-catenin pathways, making PBE a potential therapeutic for this type of cancer and an appropriate candidate for in vivo models and future clinical trials.

## Methods

### Plant material, extraction preparation, and analytical conditions

PBE was prepared by the Natural Products Research laboratory of the School of Pharmacy at the National Defense Medical Center, Taiwan. Briefly, 50.5 g dry plant of* P. barbatum* was ground into a fine powder and soaked with 4L 95% EtOH (3 times). The solvent was evaporated under reduced pressure to yield 5.5371 g PBE. HPLC analysis of PBE and quercetin was performed on a Hitachi L-7100 instrument with a UV detector (L-2400), pump (L-2130), and autosampler (L-2200). Reverse-phase separation of the marker compound was performed using a Lichro CART^®^ RP-8 (4.0 × 250 mm i.d., 5 μm) column and a gradient elution was achieved using two solvents, namely water (A) and methanol (B), at a flow rate of 1 mL/min. The gradient program consisted of an initial linear increase from 40% B to 60% B over 15 min, followed by an increase to 80% B over 15 min, and an linear increase from 80 to 100% B over 10 min. The injection volume was 10 μL and the UV absorption spectra were recorded online at 254 nm, then the data were processed using the Hitachi Model D-2000 Elite Chromatography Data Station Software.

### Cell culture and viability assays

Human colon cancer cell lines HCT-116 (p.Gly13Asp), HT-29 (p.Val600Glu) and HDF cells were obtained from the American Type Culture Collection (ATCC, Manassas, VA, USA). DPSC cells were from Lonza (Lonza, Walkersville, MD, USA). HCT-116 and HT-29 cells cultured in RPMI-1640 medium with 10% fetal bovine serum (FBS) at 37 °C. DPSCs were cultured with DPSC Basal Medium (Lonza, Walkersville, MD, USA), supplemented with DPSCGM SingleQuots Kit (Lonza). HDF cells were cutured in FibroLife basal media (Lifeline Cell Technology, Walkersville, MD, USA) with 10% FBS. Cell viability was assayed using a Cell Counting kit-8 according to the manufacturer’s instructions (CCK-8, Dojindo, Japan)^45^.

### Colony formation assay

This assay was completed as previously described^46^. Briefly, cells were incubated for 14 d at 37 °C in RPMI-1640 medium containing 10% FBS, 1 mM glutamine, 100 units penicillin, and 100 μg/mL streptomycin before the colonies were counted and quantified using ImageJ software version 1.50a (National Institutes of Health, Bethesda, MD, USA).

### Migration and invasion assays

These assays were performed using 8-μm Transwell cell culture chambers, as previously described^47^. The Transwell inserts were coated with Matrigel (BD Biosciences, Bedford, MA, USA) prior to the invasion assay, but not the migration assay, and cells were seeded in the upper chamber and incubated in serum-free RPMI-1640 medium while the lower chamber was filled with medium supplemented with 10% FBS. Cells were added to the upper chamber and incubated for 24 h before being fixed in methanol and stained with crystal violet for 15 min. Cells at the bottom of the inserts were then counted using an inverted microscope.

### Scratch assay

These assays were completed as previously described^48^. Briefly, HCT-116 and HT-29 cells were scratched using a 100 μL pipette tip, then washed with PBS and incubated with vehicle or PBE. Wound healing was imaged using photomicrography at various time points (Leica Microsystems, Wetzlar, Germany).

### Flow cytometry

Cellular apoptosis was analyzed using an Annexin-V/7-AAD staining kit according to the manufacturer’s instructions (BioVision, Inc., CA, USA). Cells were treated with vehicle or PBE and then stained with Annexin-V/7-AAD solution and analyzed using a flow cytometer (BD Biosciences, FACS CaliburTM)^46^.

### Nuclear extracts and western blotting

These assays were completed by using RIPA lysis buffer as previously described^49^. The details for the primary and secondary antibodies used in these assays are summarized in supplemet Table 5. The blots were developed using an enhanced chemiluminescence kit (Amersham Biosciences, Buckinghamshire, UK) and measured using a luminescent image analyzer (LAS-3000; Fuji Photo Film Co., Ltd., Tokyo, Japan). The membrane images were accurately spliced based on the molecular weight of each protein target, ensuring the scientific integrity of the data. This splicing occurred before the antibody hybridization step to enable the simultaneous probing of multiple target proteins on a single blot.

We assessed multiple target proteins on the same blots, which required cropping the membranes. This cropping was necessary to efficiently present data for all target proteins within the constraints of space and clarity in the figures and for all replicates performed in the Supplementary Information file.

### Co-immunoprecipitation assays

These assays were performed as previously described^41^. Briefly, cells were lysed and incubated with anti-YAP beads for 4 h at 4 °C, then washed before the immunocomplexes were suspended in SDS sample buffer and subjected to western blotting. The inputs were loaded with total cell lysates for comparison.

### RNA sequencing

An RNA sequencing library was prepared using the TruSeq Stranded mRNA Library Prep Kit (Illumina, San Diego, CA, USA) and sequenced on an Illumina NovaSeq 6000 platform (150 bp paired-end reads) run by the Genomics, BioSci & Tech Co., New Taipei City, Taiwan. The quality of the libraries was assessed using an Agilent Bioanalyzer 2100 system and real-time PCR. The reads were mapped to the reference genome using Bowtie2 (version 2.3.4.1)^50^. Transcript abundance was quantified using RSEM (version 1.2.28)^51^ and DEGs were identified using EBSeq (version 1.16.0)^52^. FPKM was caculated for the gene expression. GO and KEGG pathway analysis were used to evaluate the gene clusters identified by the clusterProfiler program in R (version 3.6.0)^53^.

### Statistical analysis

All data are presented as the mean ± standard deviation of the mean (SD) and the differences between groups were evaluated using one-way analysis of variance (ANOVA) and a Bonferroni post-hoc test. Statistical analyses were performed using IBM SPSS Statistics version 22 (IBM^®^ SPSS^®^ Statistics 22) and significance was accepted when the p values were less than 0.05.

### Ethics approval and consent to participate

All cell lines used in our study were commercialized cell lines and were purchased from American Type Culture Collection (ATCC, Manassas, VA, USA) and Lonza (Lonza, Walkersville, MD, USA). All the methods used in this study were performed in accordance with relevant guidelines and regulation as approved by the research ethics committee. *Polygonum barbatum* is not under protection in Taiwan. All methods were in compliance with relevant institutional, national, and international guidelines and legislation.

### Supplementary Information

Supplementary Information.

## Data Availability

The datasets generated and analyzed during the current study are available from the corresponding author on reasonable request.
