# Supplementary material for: Polygonum barbatum extract reduces colorectal cancer cell proliferation, migration, invasion, and epithelial–mesenchymal transition via YAP and β-catenin pathway regulation
Source: Sci Rep. 2023 Oct 26;13:18368. doi: 10.1038/s41598-023-45630-1 (PMC10603200; doi:10.1038/s41598-023-45630-1)

*Polygonum barbatum* extract reduces colorectal cancer cell proliferation, migration, invasion, and epithelial-mesenchymal transition via YAP and β-catenin pathway regulation

Pi-Kai Chang, I-Chuan Yen, Wei-Cheng Tsai, Shih-Yu Lee

**Supplementary information**

**Supplementary Table 1.** Quality and read statistics for the RNA sequencing data.

| **Sample** | **Clean data size (bp)** | **Clean reads number** | **Q30 (%)** | **Total mapped reads (%)** |
| --- | --- | --- | --- | --- |
| Control | 6,193,238,770 | 21,901,128 | 95.37% | 77.36% |
| PBE 30 | 4,756,679,235 | 17,279,188 | 95.10% | 75.01% |
| PBE 100 | 5,637,303,528 | 20,925,391 | 95.13% | 73.17% |
| 5-FU | 5,799,469,908 | 20,315,972 | 95.19% | 74.53% |

PBE, *Polygonum barbatum* extract; 5-FU, 5-fluorouracil.

**Supplementary Table 2.** KEGG pathway enrichment of the top 10 molecular pathways of DEGs in the 30 μg/mL PBE group as identified by RNA sequencing.

| **KEGG ID** | **Description** | **Gene Ratio** | ***P* value** |
| --- | --- | --- | --- |
| hsa04512 | ECM–receptor interaction | 5/50 | 0.000200515 |
| hsa05144 | Malaria | 4/50 | 0.000246013 |
| hsa05143 | African trypanosomiasis | 3/50 | 0.001511117 |

KEGG, Kyoto Encyclopedia of Genes and Genomes; DEGs, differentially expressed genes.

**Supplementary Table 3.** KEGG pathway enrichment of the top 10 molecular pathways of DEGs in the 100 μg/mL PBE group as identified by RNA sequencing.

| **KEGG ID** | **Description** | **Gene Ratio** | ***P* value** |
| --- | --- | --- | --- |
| hsa04512 | ECM–receptor interaction | 13/113 | 2.0363E-10 |
| hsa04933 | AGE–RAGE signaling pathway in diabetic complications | 7/113 | 0.000487805 |
| hsa04510 | Focal adhesion | 10/113 | 0.000500723 |
| hsa05165 | Human papillomavirus infection | 13/113 | 0.000711372 |
| hsa04151 | P13K–Akt signaling pathway | 13/113 | 0.000132048 |
| hsa05222 | Small cell lung cancer | 6/113 | 0.0001794871 |
| hsa04974 | Protein digestion and absorption | 6/113 | 0.0002114667 |
| hsa05031 | Amphetamine addiction | 5/113 | 0.0002746707 |

**Supplementary Table 4.** KEGG pathway enrichment of the top 10 molecular pathways of DEGs in the 5-FU-treated group as identified by RNA sequencing.

| **KEGG ID** | **Description** | **Gene Ratio** | ***P* value** |
| --- | --- | --- | --- |
| hsa04390 | Hippo signaling pathway | 21/188 | 7.11685E-11 |
| hsa04510 | Focal adhesion | 20/188 | 3.85421E-08 |
| hsa05205 | Proteoglycans in cancer | 20/188 | 5.37371E-08 |
| hsa04010 | MAPK signaling pathway | 24/188 | 7.43009E-08 |
| hsa05219 | Bladder cancer | 9/188 | 3.14295E-07 |
| hsa05210 | Colorectal cancer | 12/188 | 6.28017E-07 |
| hsa04115 | p53 signaling pathway | 10/188 | 6.59901E-06 |
| hsa05225 | Hepatocellular carcinoma | 15/188 | 8.11604E-06 |
| hsa04810 | Regulation of actin cytoskeleton | 17/188 | 9.65181E-06 |
| hsa05224 | Breast cancer | 13/188 | 3.67231E-05 |

**Supplementary Table 5.** Antibodies used for Western blotting

| Type | Antigen | Manufacturer | Dilution |
| --- | --- | --- | --- |
| Primary antibody | ZO-1 | Proteintech, catalogue number 21773-1-AP | 1:4000 |
| Primary antibody | E-cadherin | GeneTex, catalogue number GTX100443 | 1:2000 |
| Primary antibody | N-cadherin | iReal Biotechnology, catalogue number IR46-143 | 1:2000 |
| Primary antibody | Vimentin | Abcam, catalogue number ab92547/EPR3776 | 1:1000 |
| Primary antibody | Slug | Cell signaling, catalogue number [#9585](https://www.cellsignal.com/products/primary-antibodies/slug-c19g7-rabbit-mab/9585?__hsfp=2027992606&__hssc=130705104.1.1620432000221&__hstc=130705104.6fa385653ecd7c9674ba06f08984886d.1620432000218.1620432000219.1620432000220.1&Ns=productCitationsCount%7C1&N=0+4294956287&Nrpp=200&No=%7Boffset%7D&fromPage=plp) | 1:500 |
| Primary antibody | Snail | GeneTex, catalogue number GTX100754 | 1:500 |
| Primary antibody | β-actin | Cell signaling, catalogue number #4970 | 1:5000 |
| Primary antibody | p-YAP 127 | Applied Biological Materials Inc. (abm), catalogue number ab76252 | 1:1000 |
| Primary antibody | YAP | Cell signaling, catalogue number #12395 | 1:1000 |
| Primary antibody | TBP | arigo Biolaboratories, catalogue number 1TBP18 | 1:1000 |
| Primary antibody | β-catenin | Cell signaling, catalogue number #9562 | 1:1000 |
| Primary antibody | GSK-3β | Cell signaling, catalogue number #9315 | 1:1000 |
| Primary antibody | Phospho-GSK-3β (Ser9) | Cell signaling, catalogue number #9336 | 1:1000 |
| Primary antibody | Phospho-β-Catenin (Ser33/37/Thr41) | Cell signaling, catalogue number #9561 | 1:1000 |
| Primary antibody | TAZ | Cell signaling, catalogue number #4883 | 1:1000 |
| Primary antibody | cyclin D1 | Cell signaling, catalogue number #2978 | 1:1000 |
| Primary antibody | Myc | Cell signaling, catalogue number #5605 | 1:1000 |
| Primary antibody | c-jun. | Cell signaling, catalogue number #9165 | 1:1000 |
| Secondary antibody | Anti-rabbit IgG-HRP | GeneTex, catalogue number GTX213110-01 | 1:10000 |
| Secondary antibody | Anti-mouse IgG-HRP | Jackson ImmunoResearch Laboratories, [111-035-003](https://shop.bio-connect.nl/antibodies/peroxidase-affinipure-goat-anti-rabbit-igg-h-l/111-035-003/sfid/1028429) | 1:10000 |

**Supplementary Figure 1a, b.** Colony formation of HT29 cells were visualized using crystal violet staining following 14 days of PBE treatment (1a). Quantitative analysis of the colony formation assay (1b), 5-FU was used as a positive control.

**Supplementary Figure 1a**

**
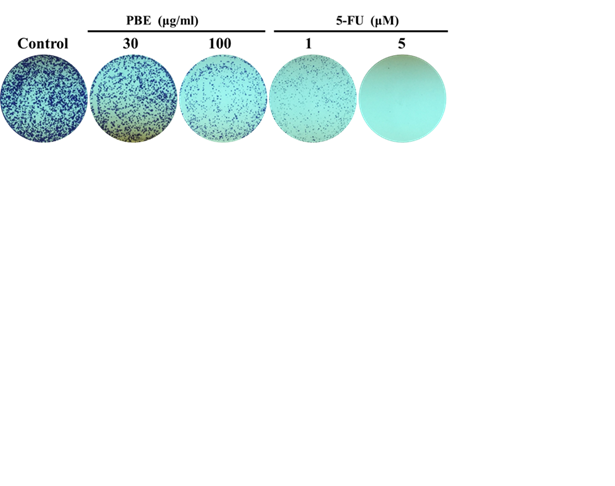
**

**Supplementary Figure 1b**

**
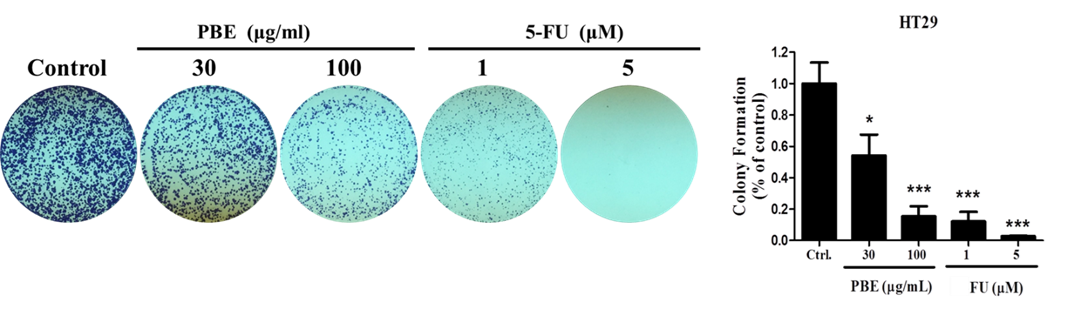
**

**Supplementary Figure 2a-i.** Gene Ontology (GO) Enrichment Analysis. Biological processes (a–c), molecular functions (d–f), and cellular components (g–i) that were most affected in PBE-treated cells compared with the control group. 5-FU was used as positive control.

**Supplementary Fig.** 2a Control vs. 30 μg/mL PBE


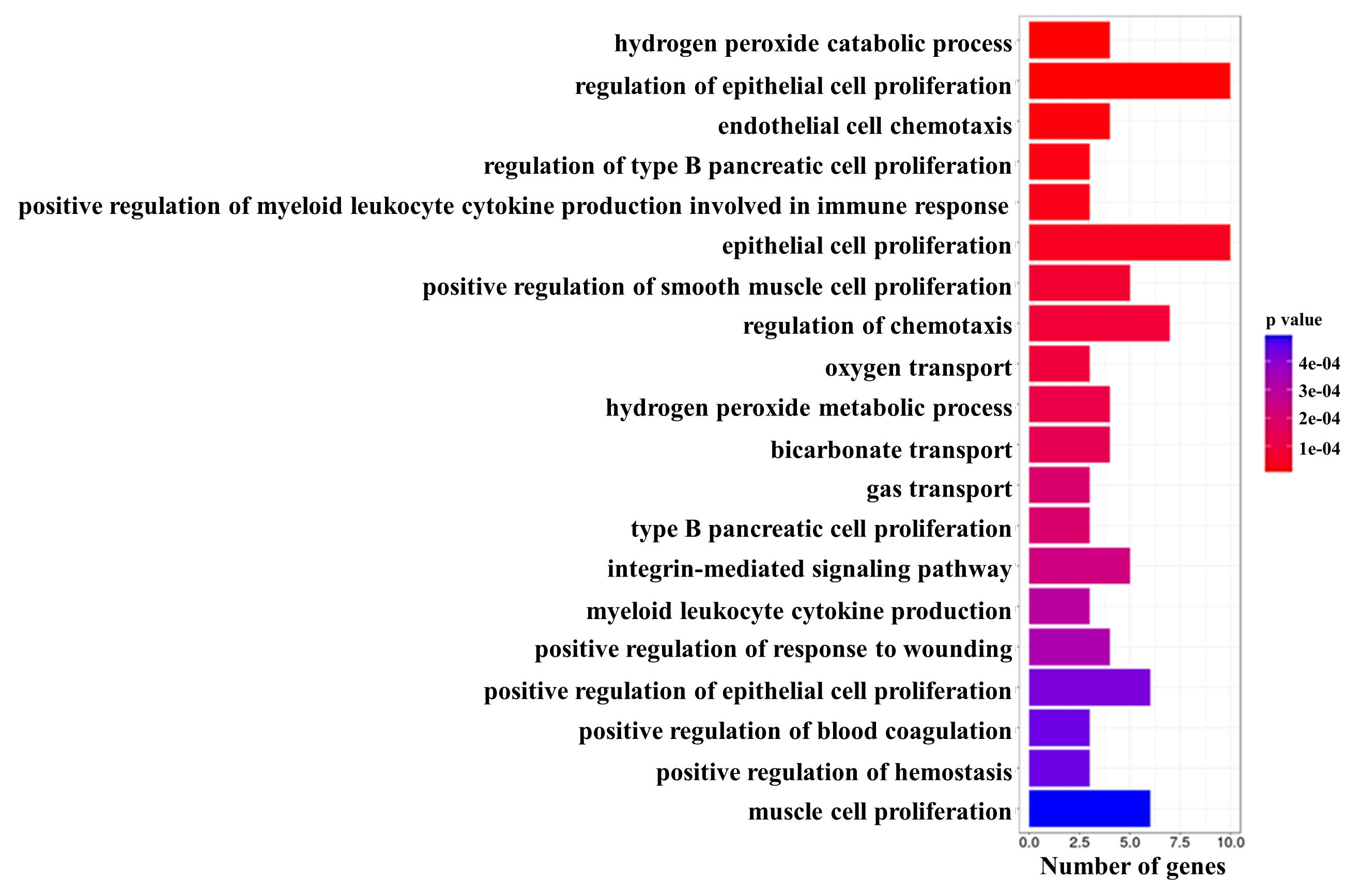


**Supplementary Fig.** 2b Control vs.100 μg/mL PBE

**
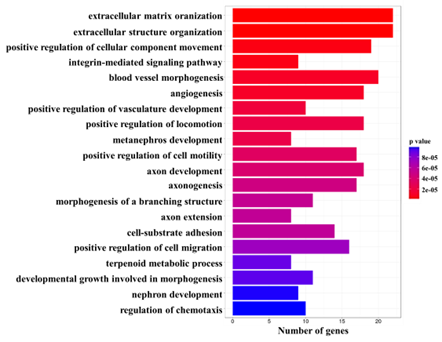
**

**Supplementary Fig.** 2c Control vs. 5-FU


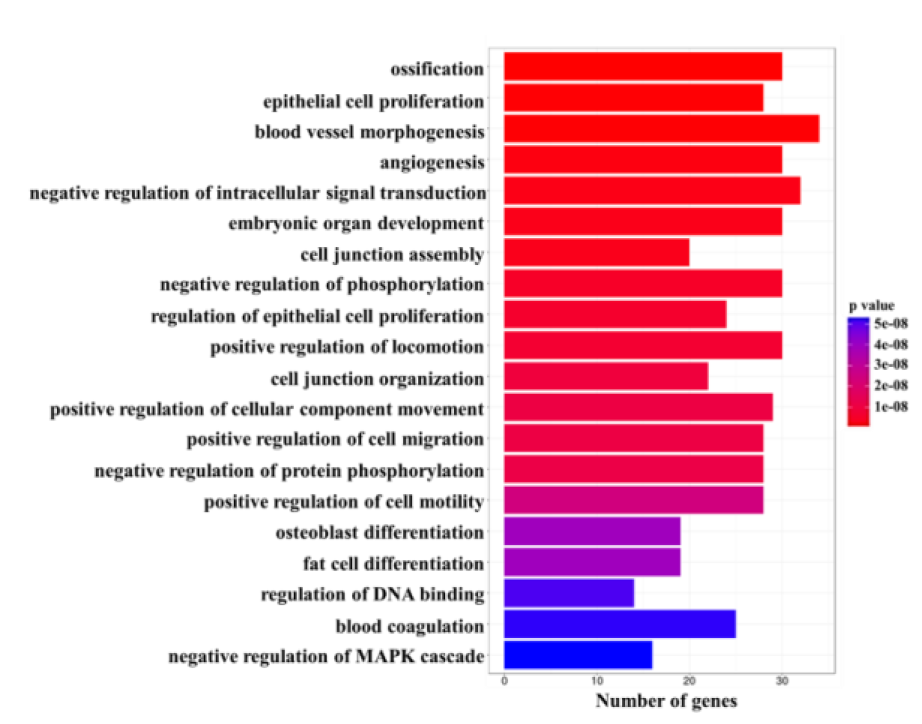


**Supplementary Fig.** 2d Control vs. 30 μg/mL PBE


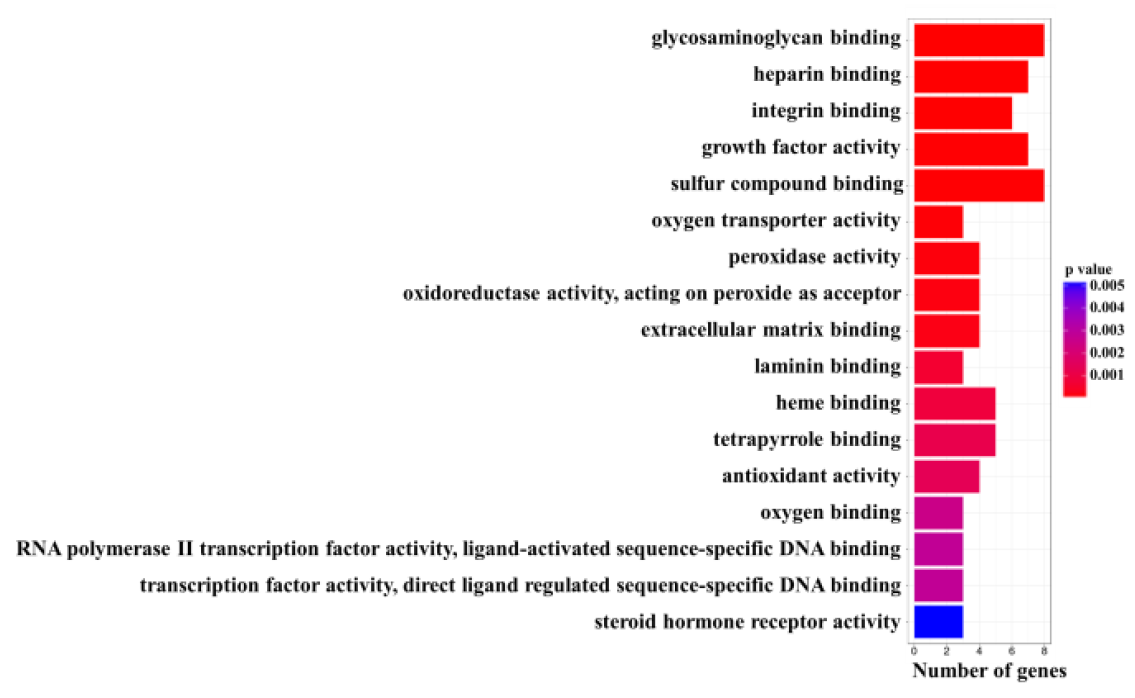


**Supplementary Fig.** 2e Control vs. 100 μg/mL PBE


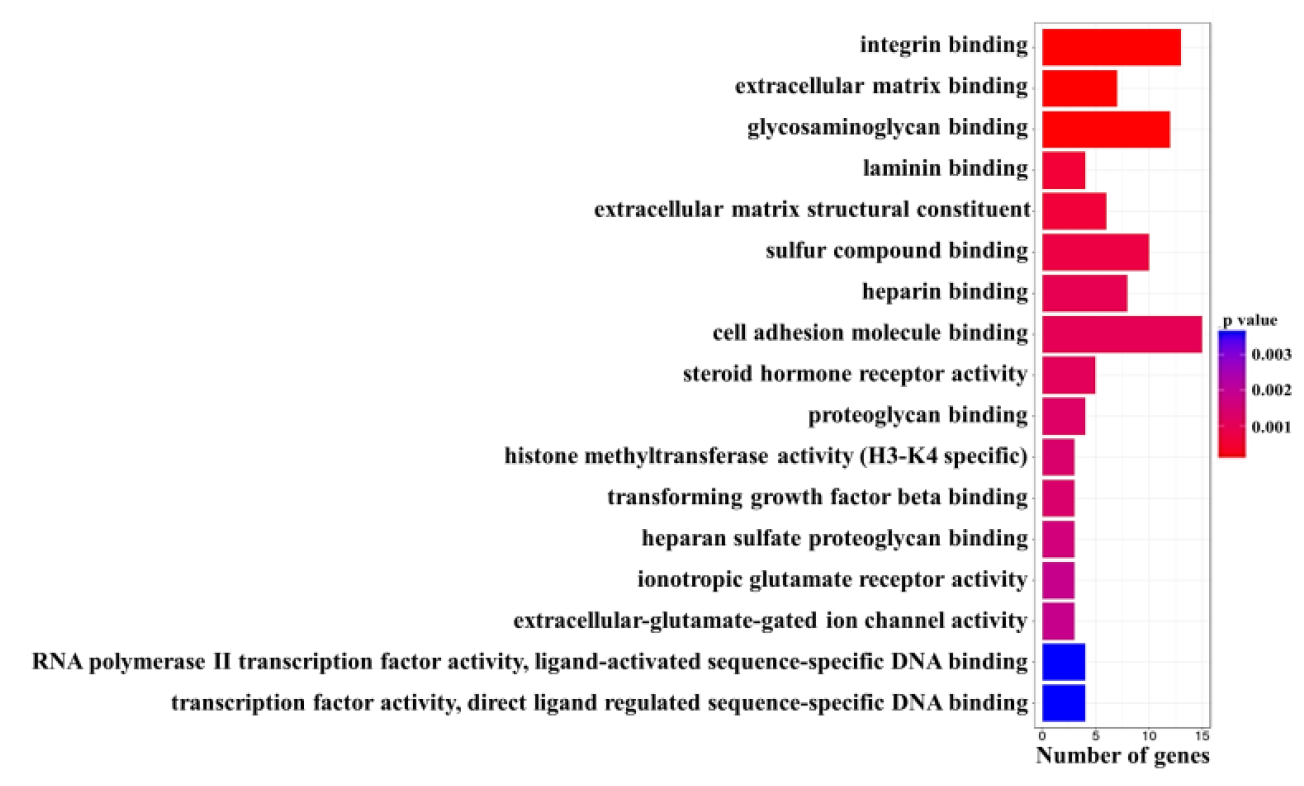


**Supplementary Fig.** 2f Control vs. 5-FU


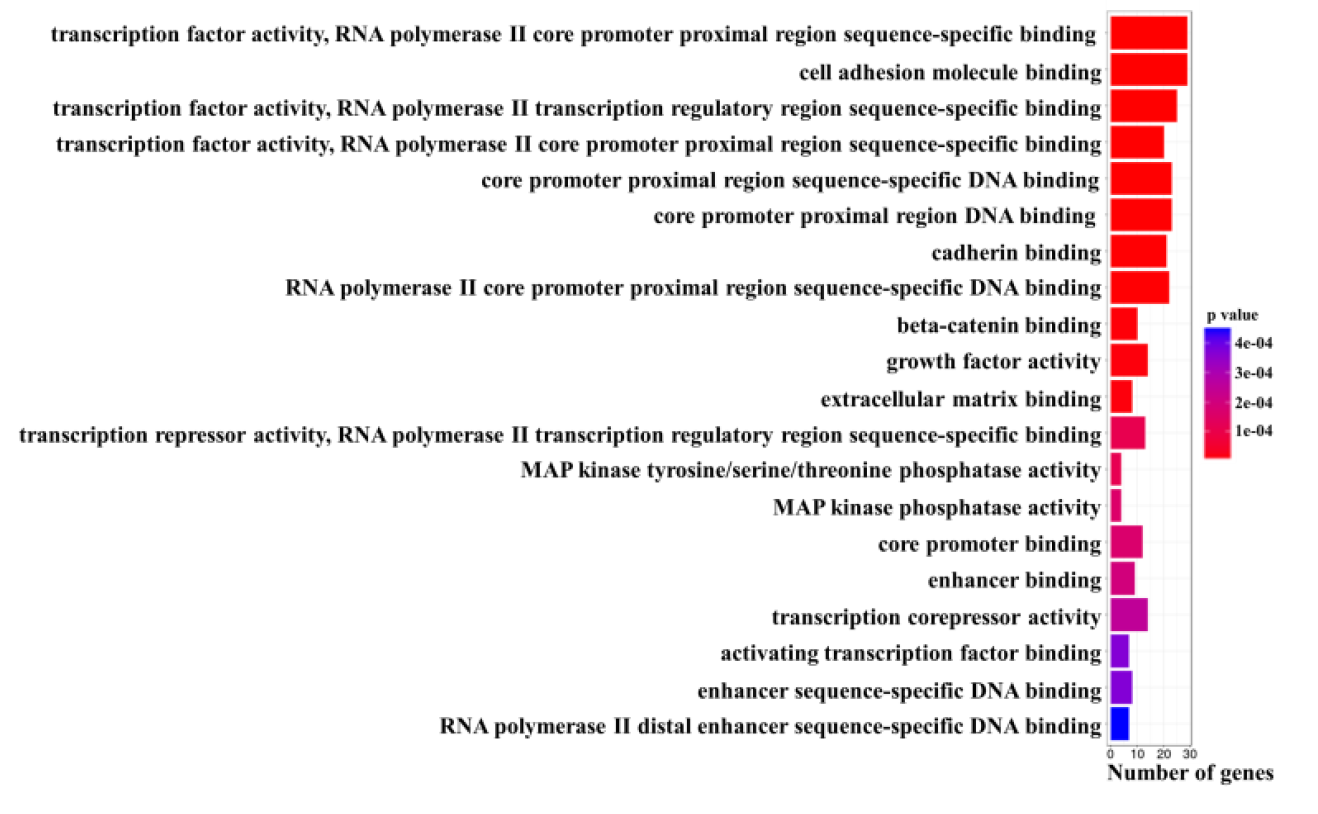


**Supplementary Fig.** 2g Control vs. 30 μg/mL PBE


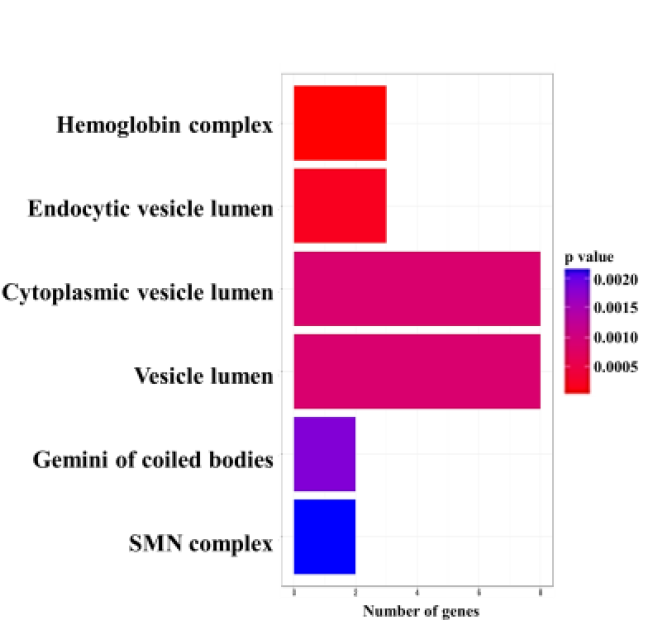


**Supplementary Fig.** 2h Control vs. 100 μg/mL PBE

**
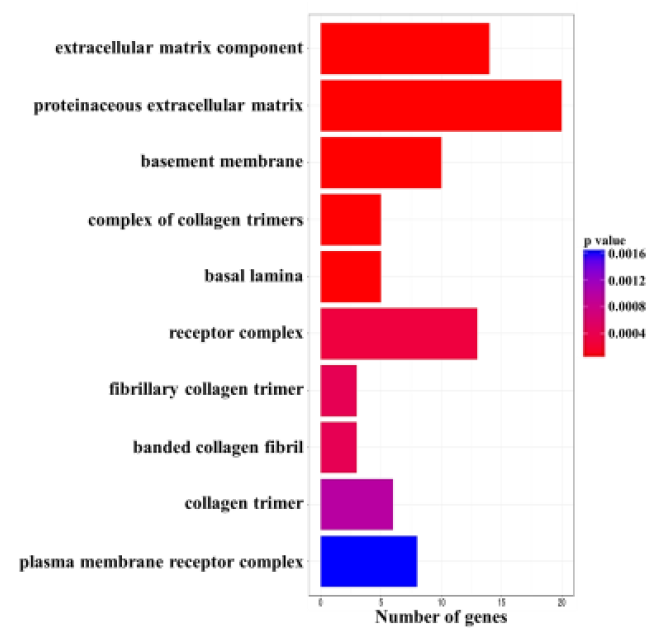
**

**Supplementary Fig.** 2i Control vs. FU


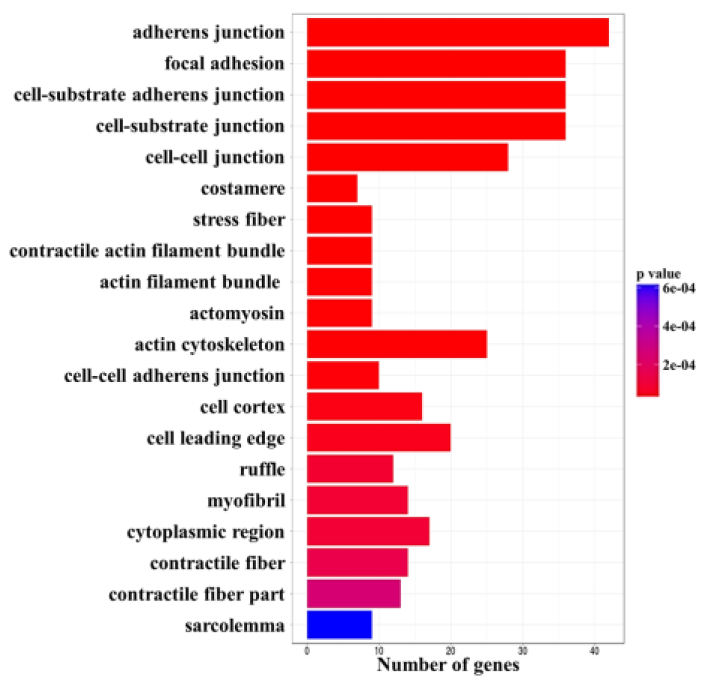


**Western blot replicates**


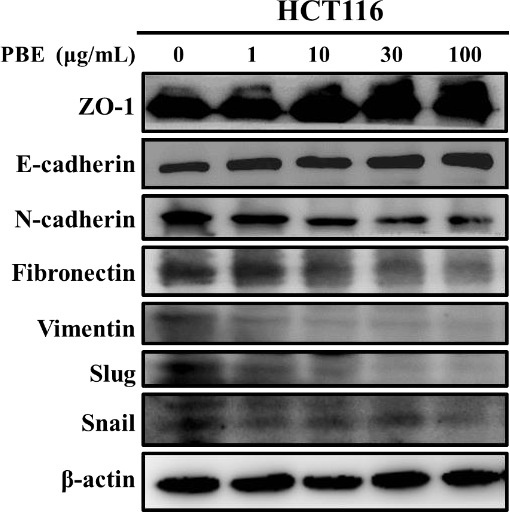


**F.5(i)**


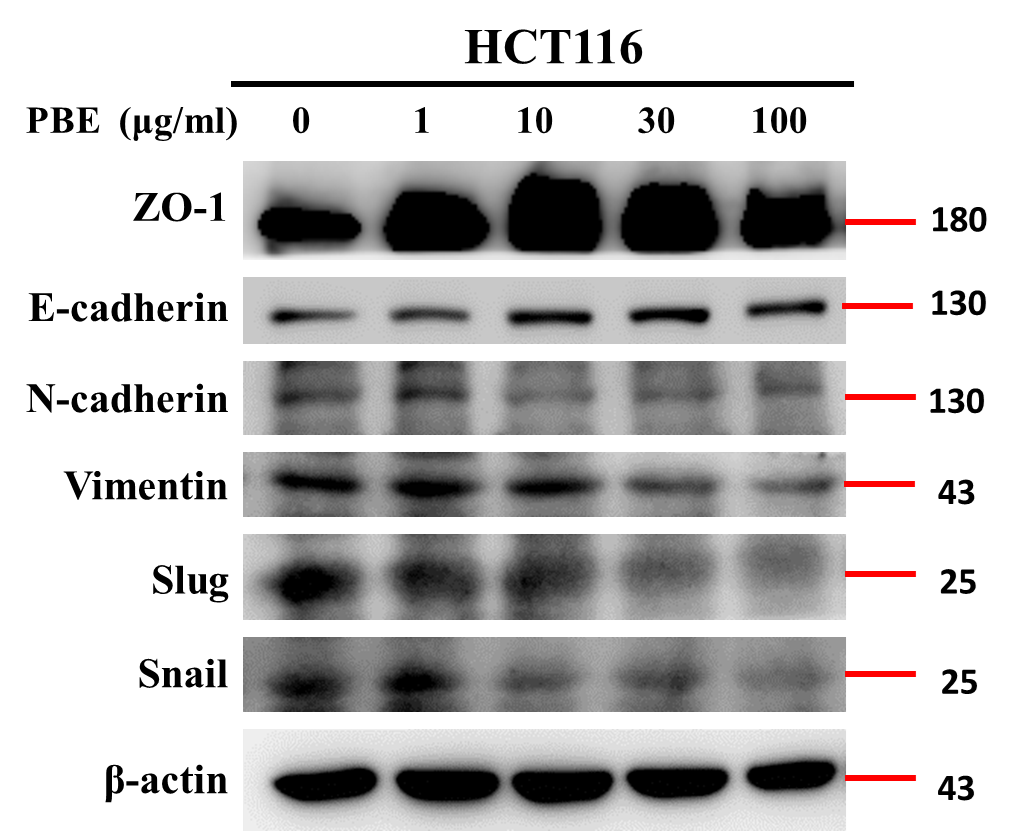


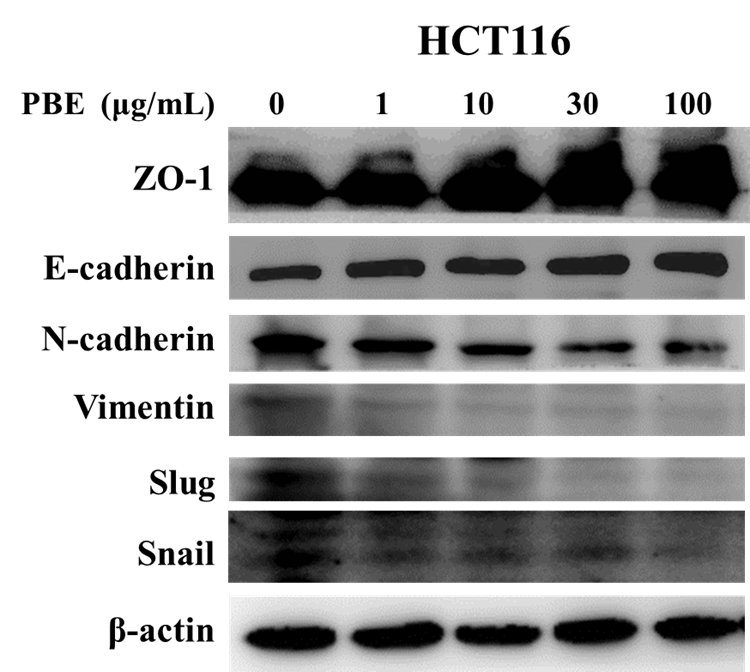


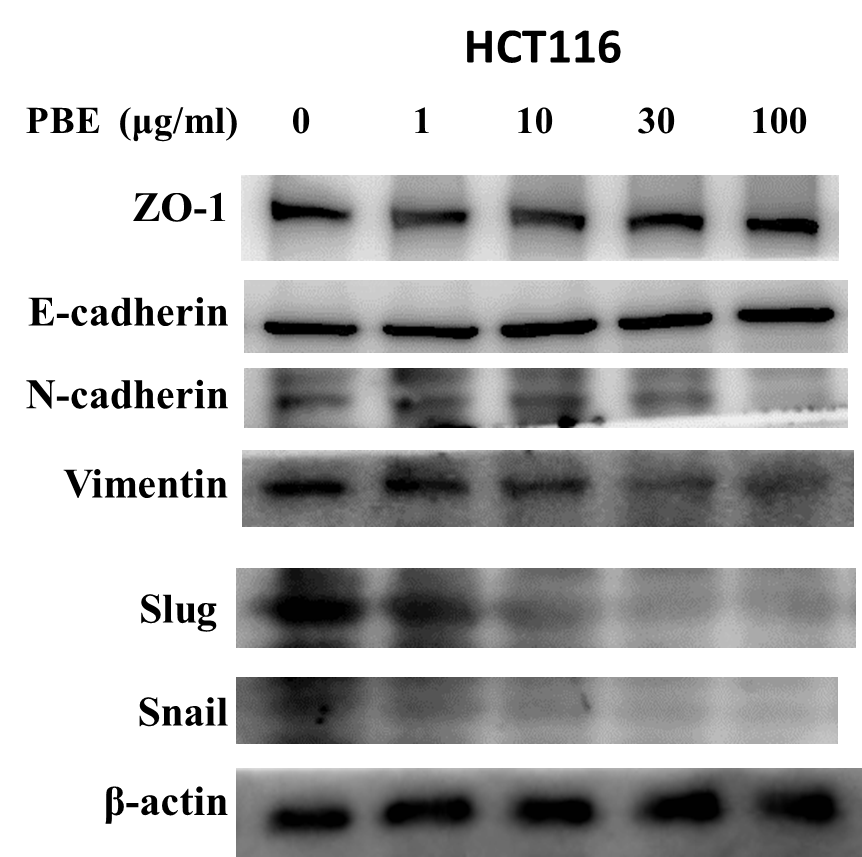


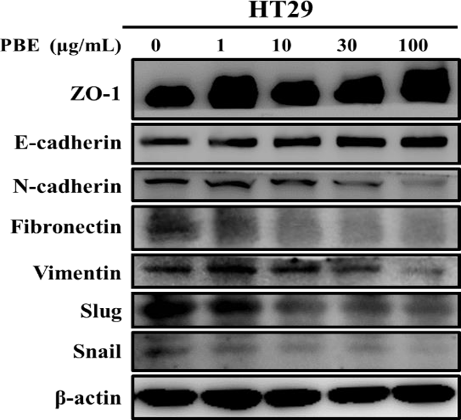


**F.5(p)**


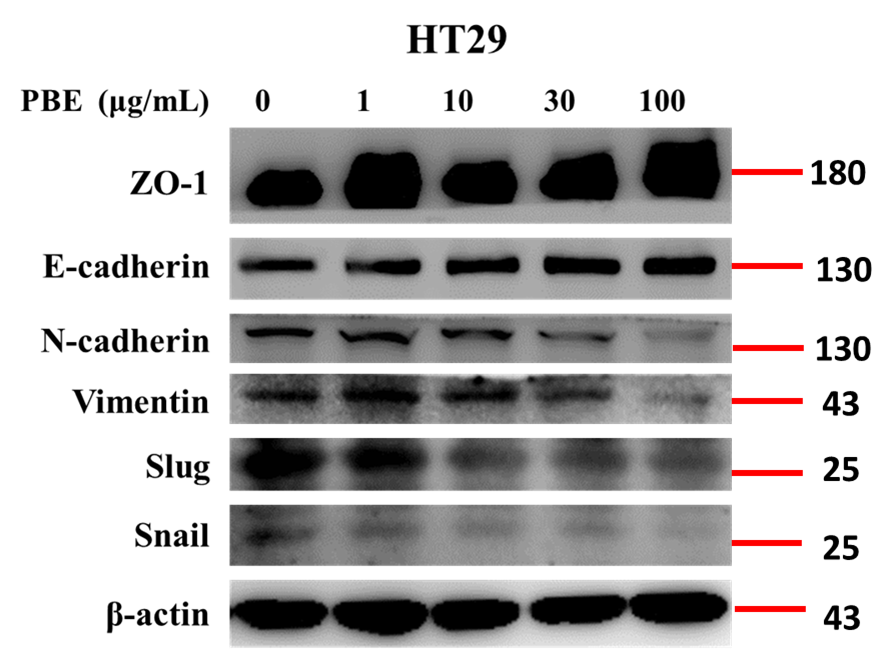


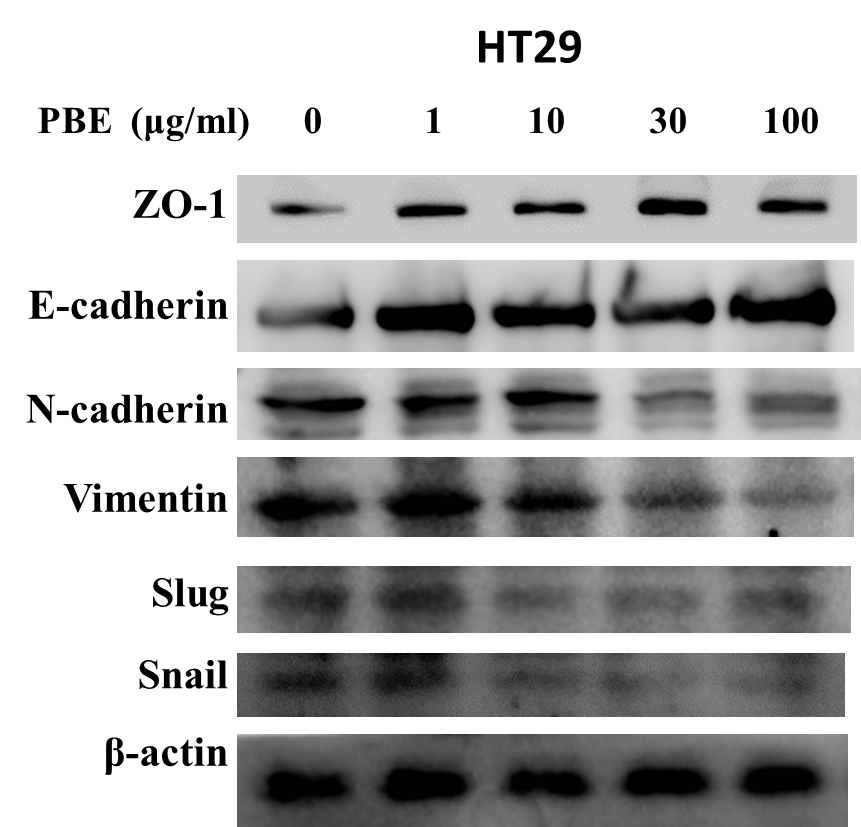


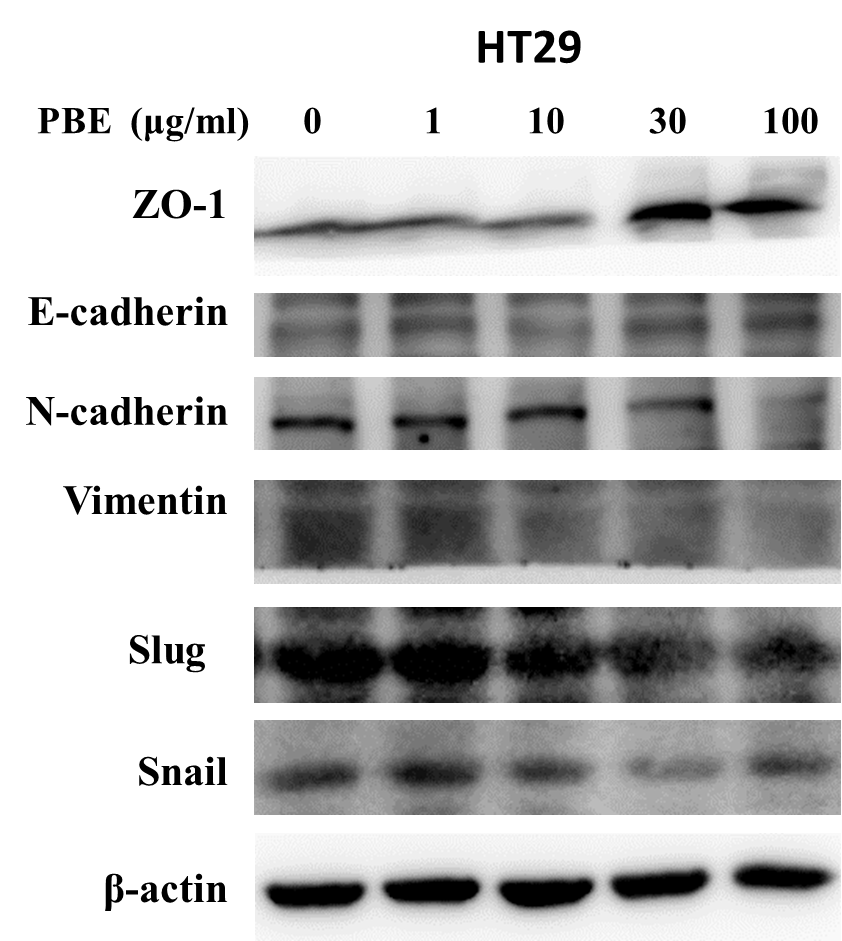


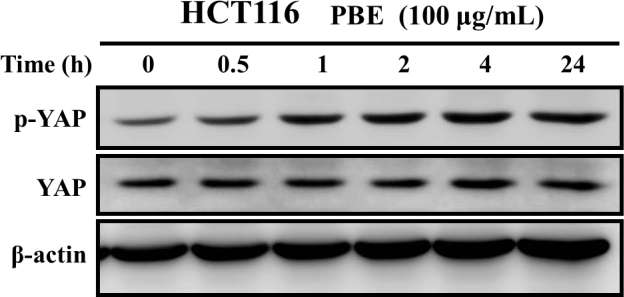


**F.6(a)**


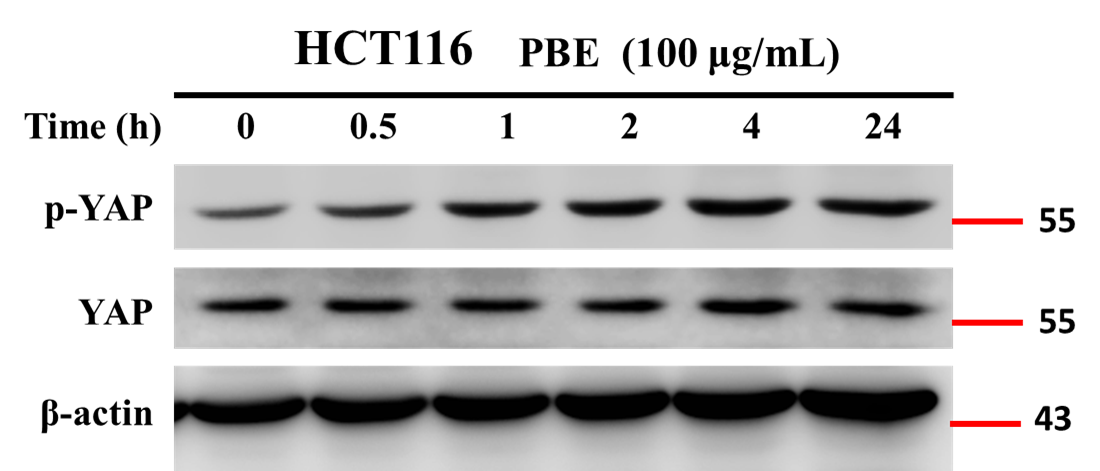


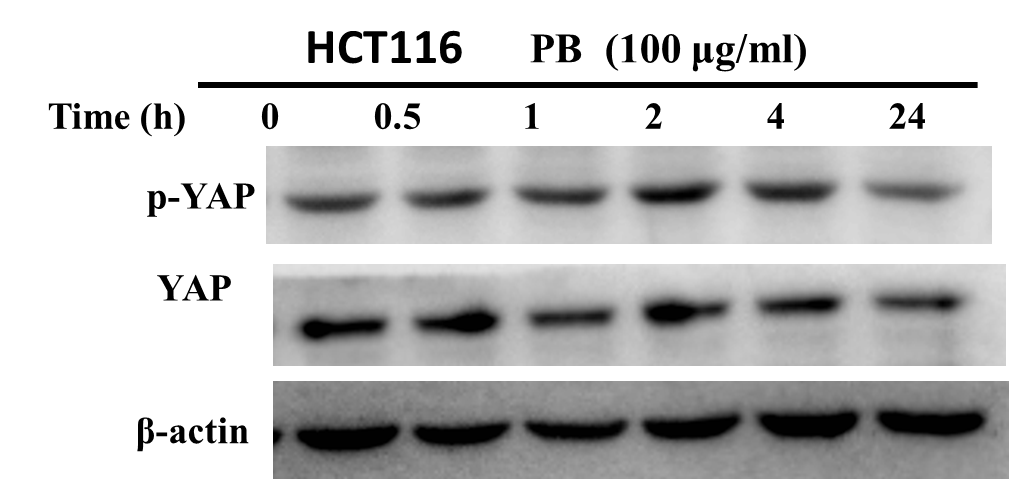


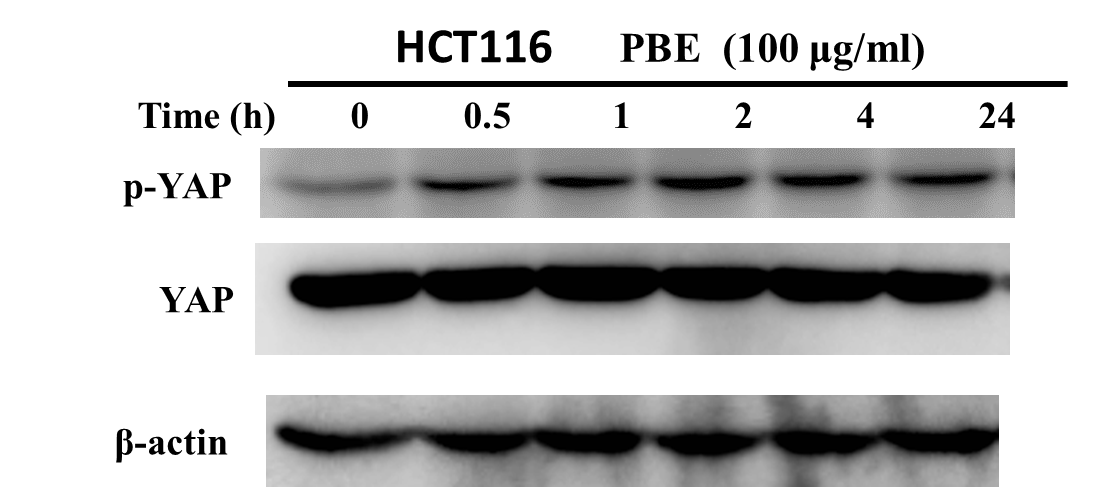


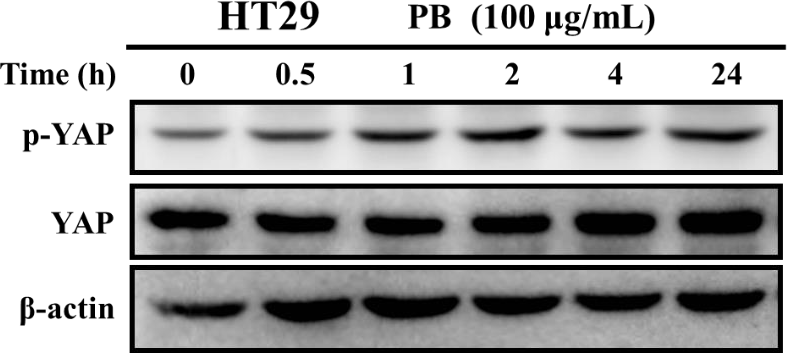


**F.6(c)**


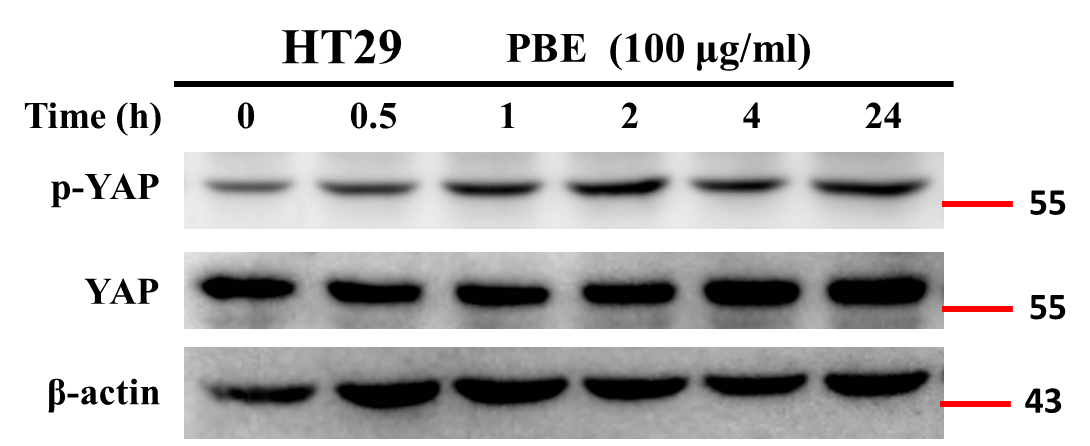


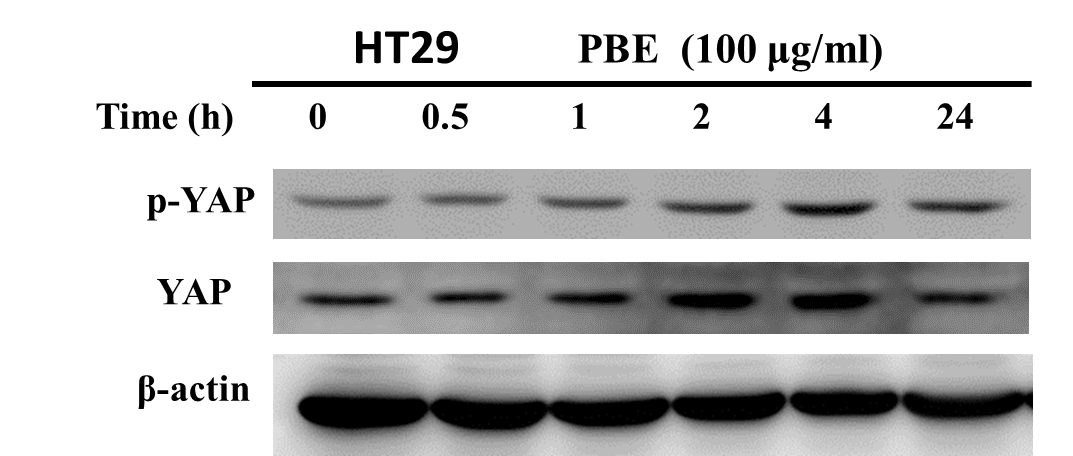


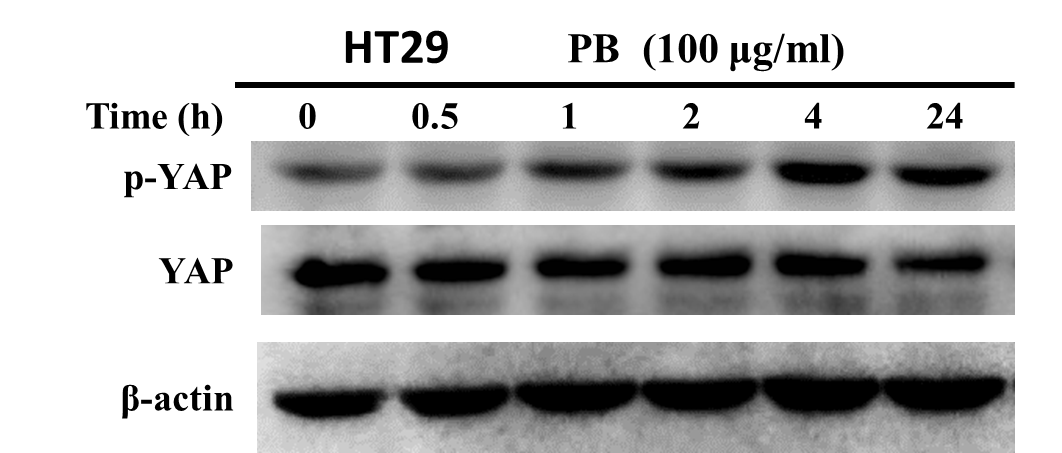


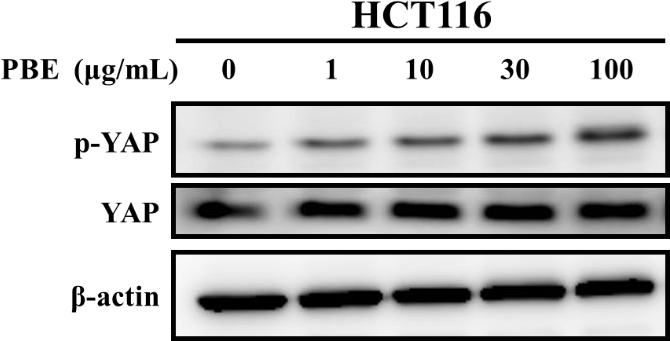


**F.6(e)**


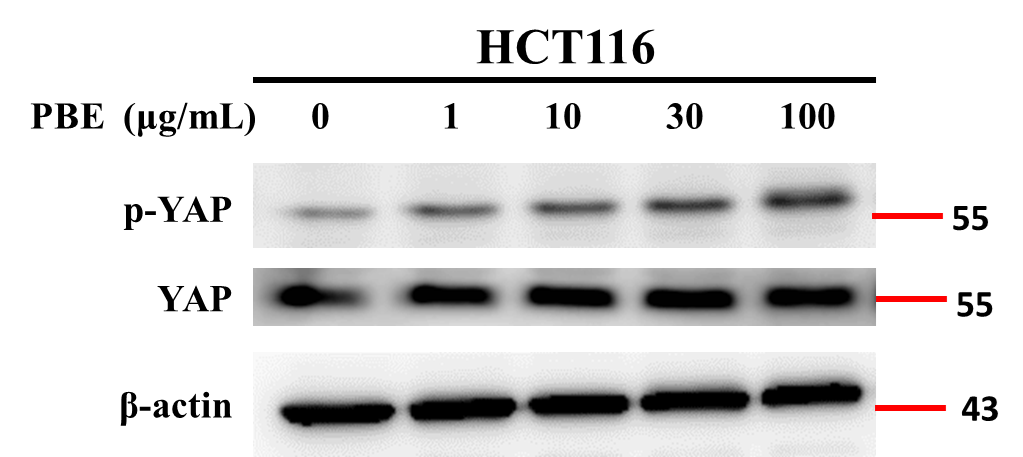


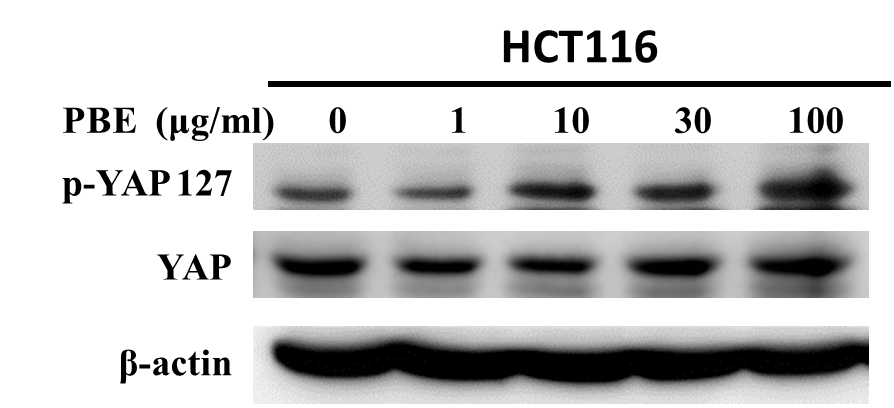


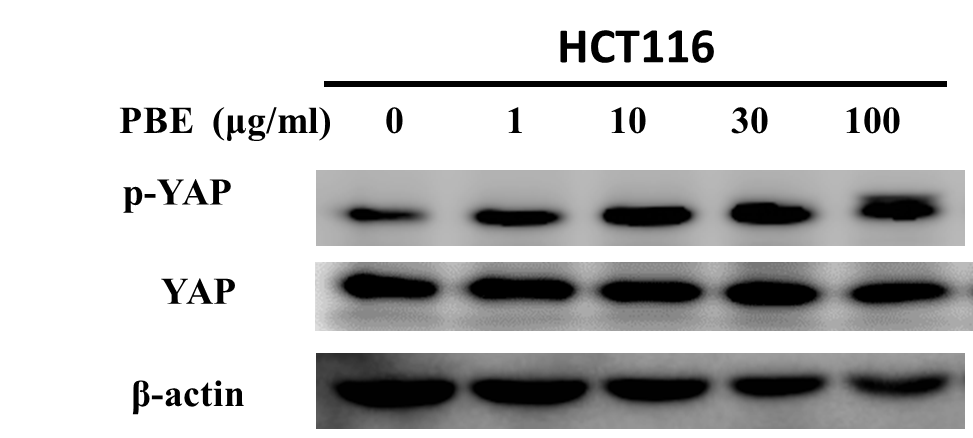


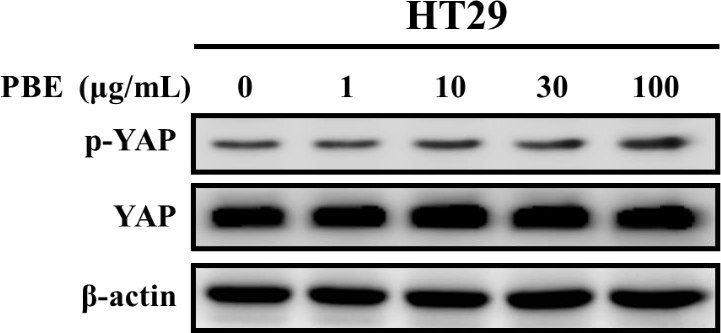


**F.6(g)**


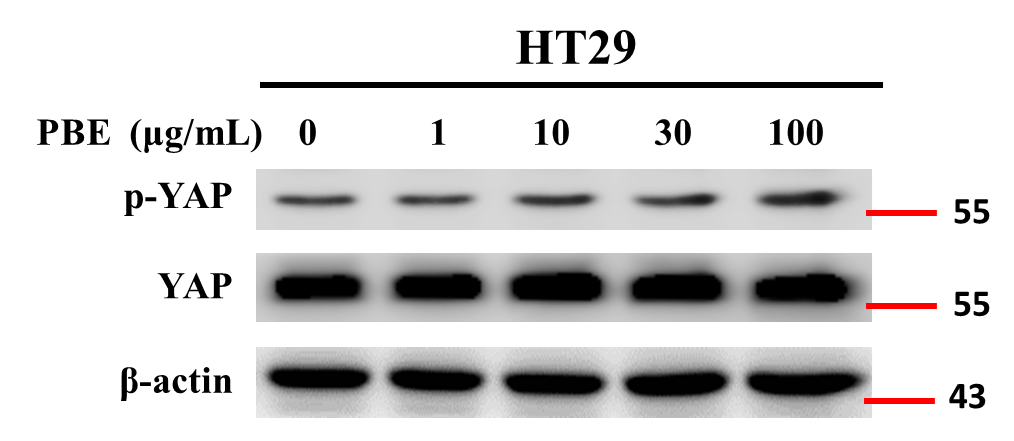


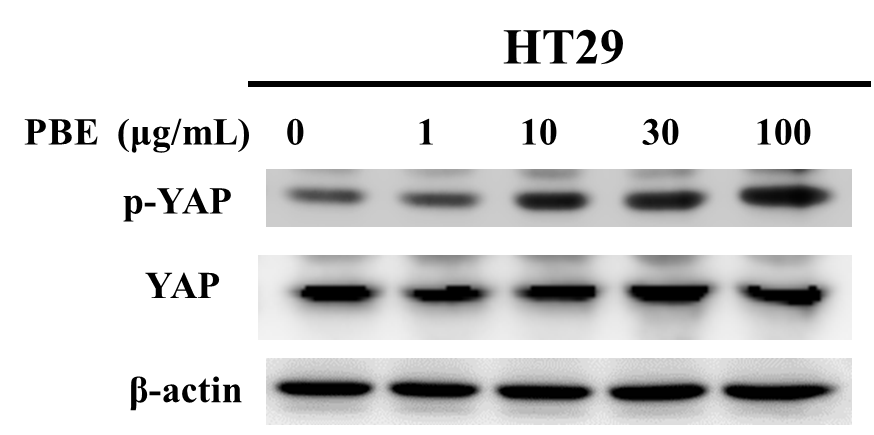


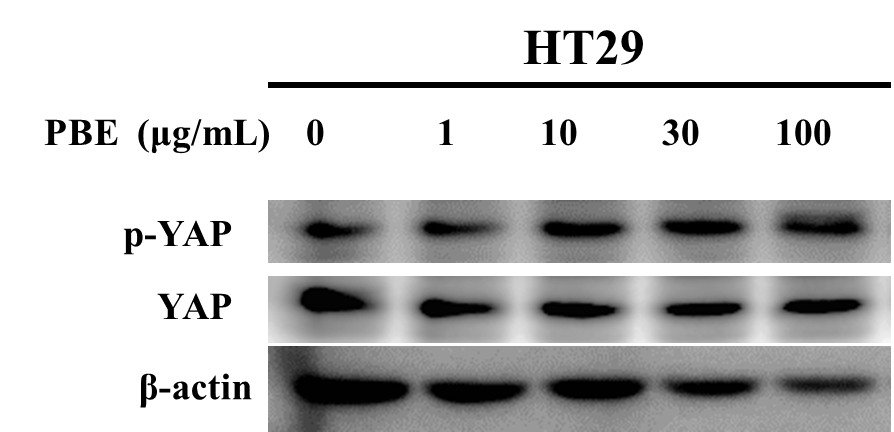


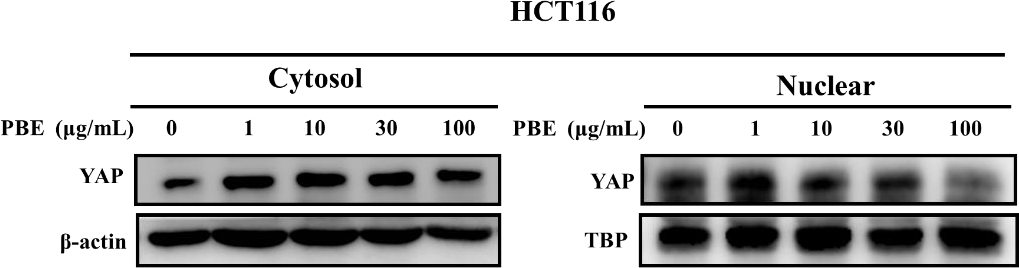


**F.6(i)**


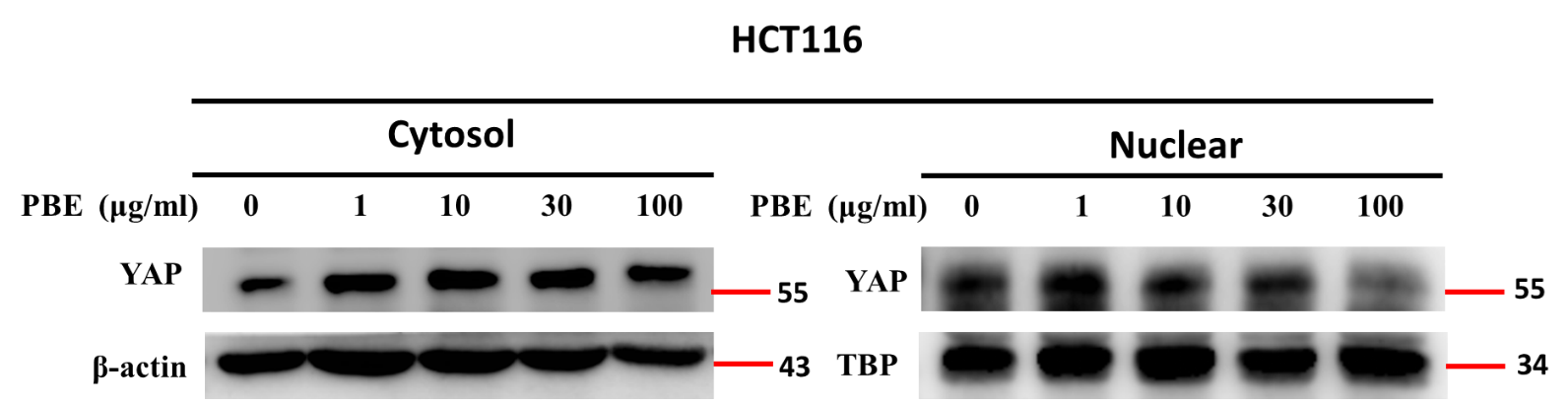


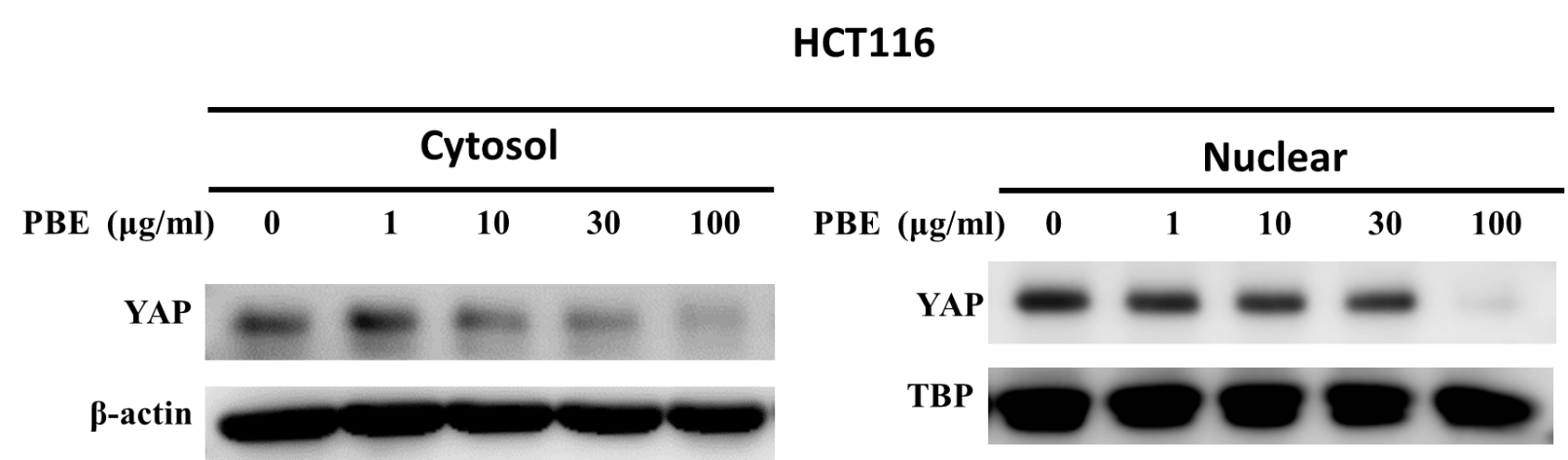


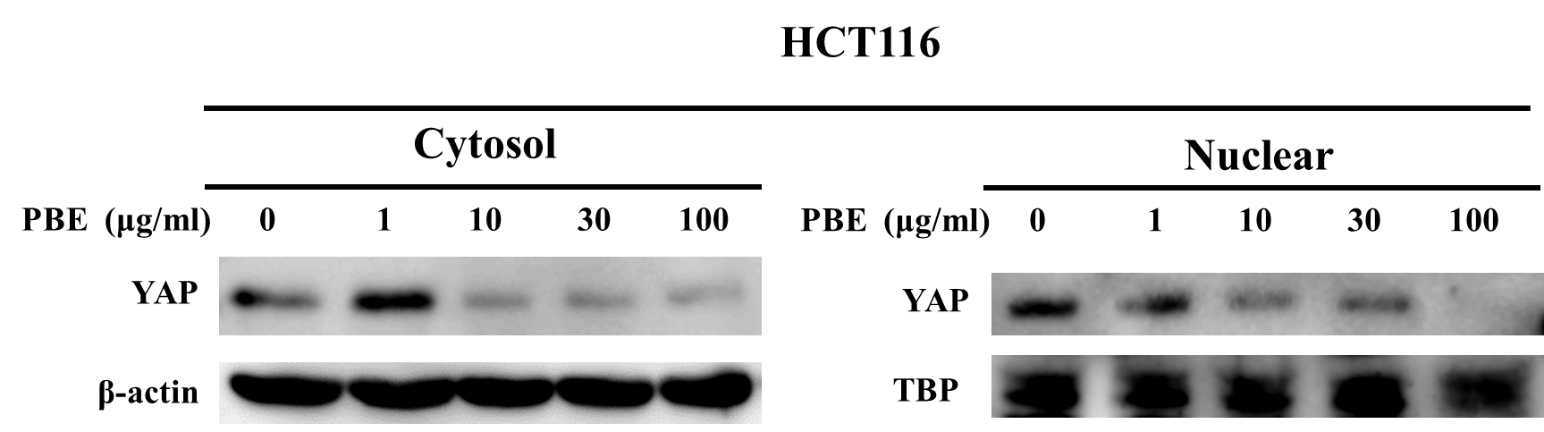


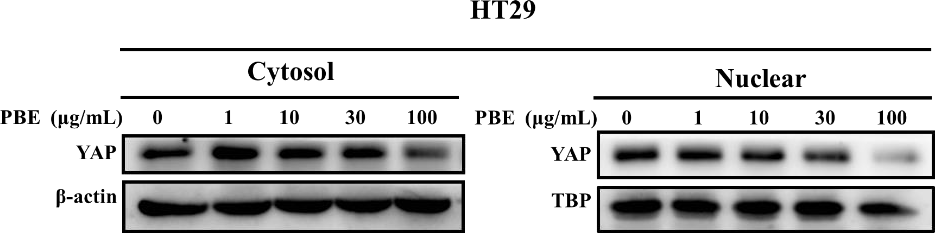


**F.6(l)**


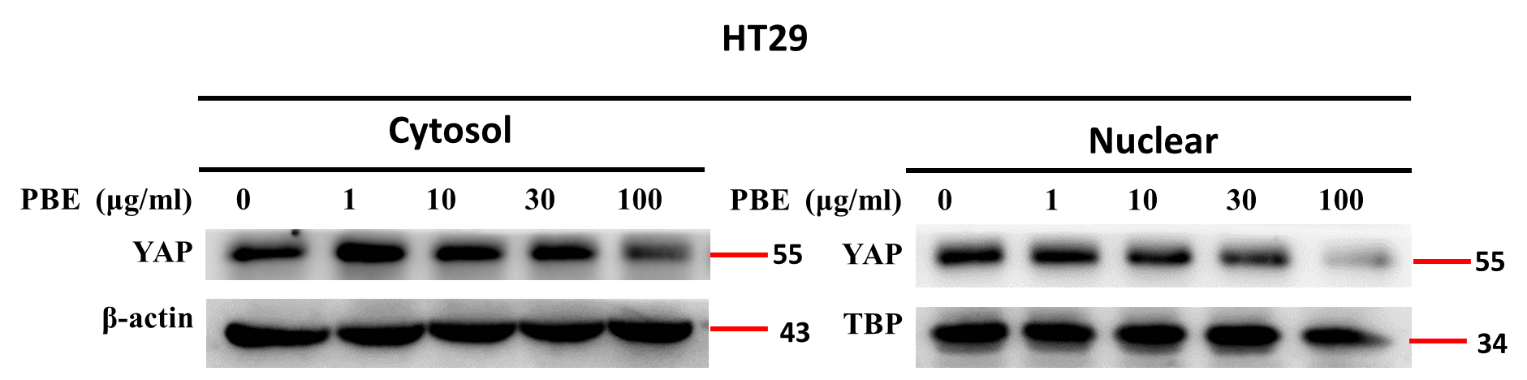


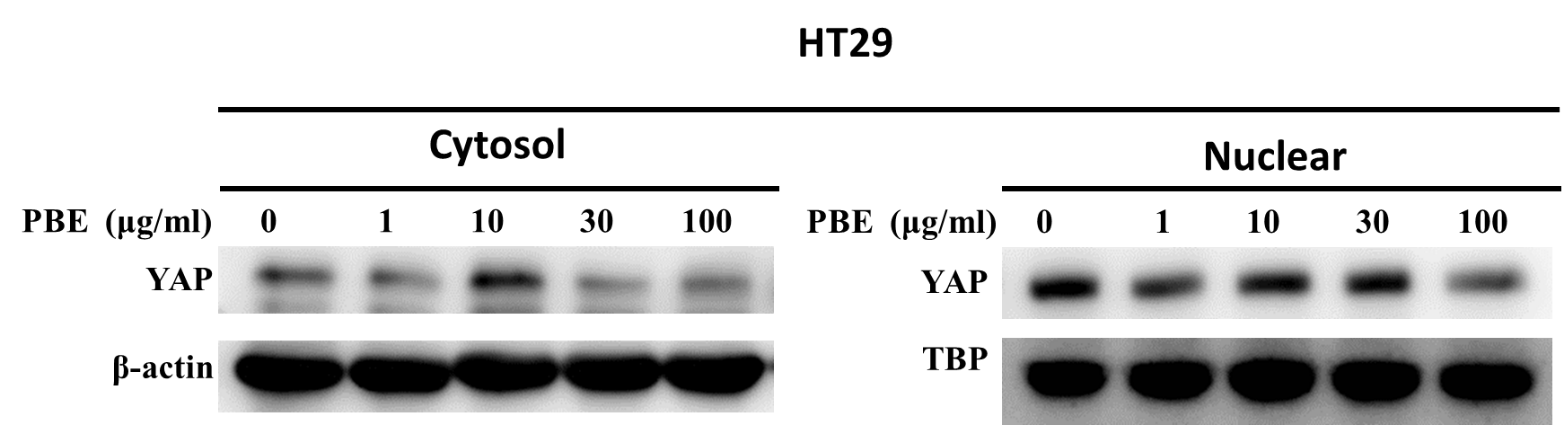


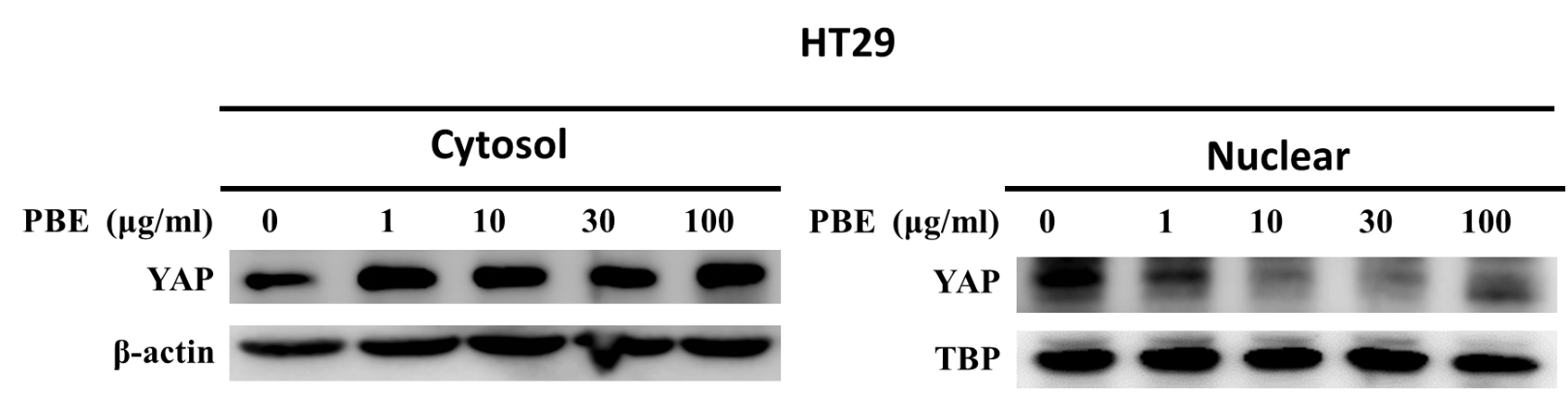


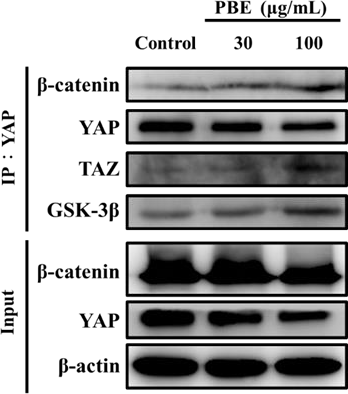


**F.6(s)**


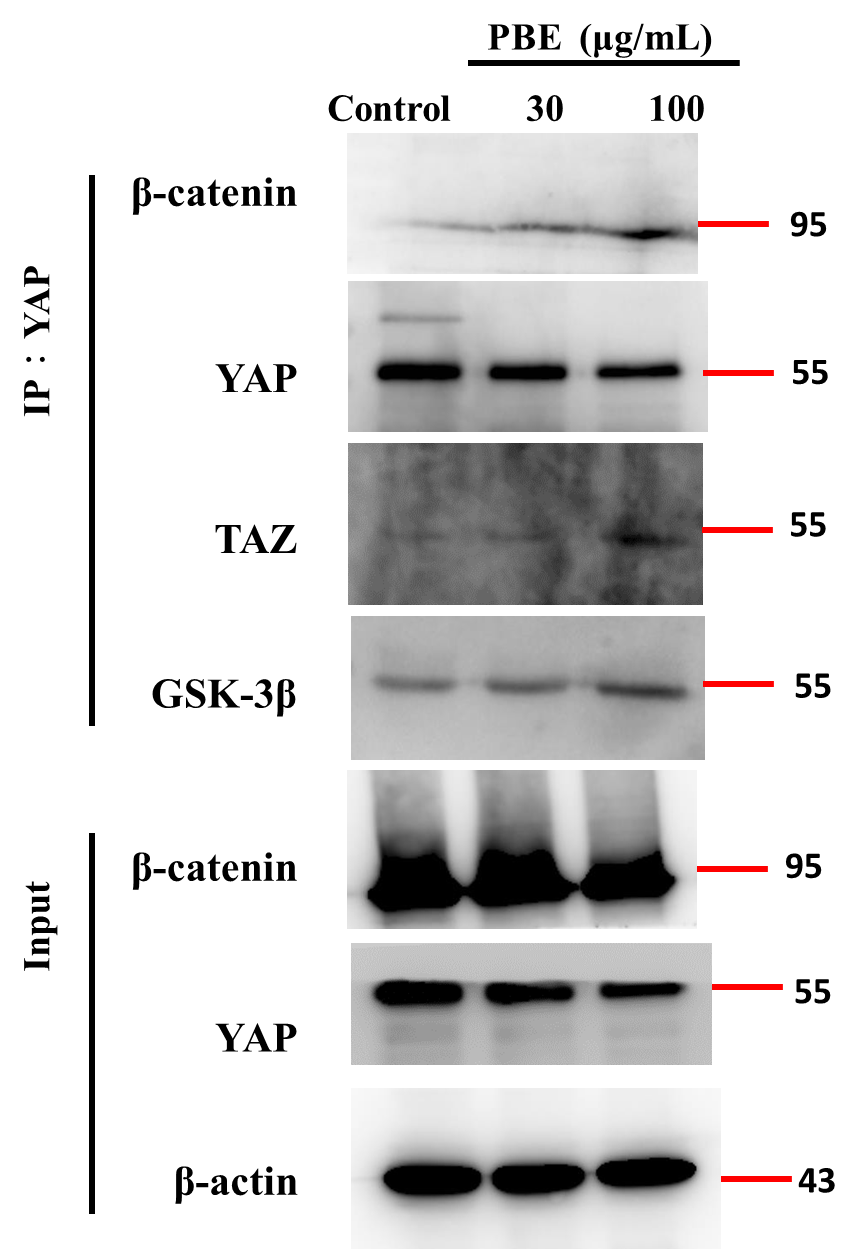


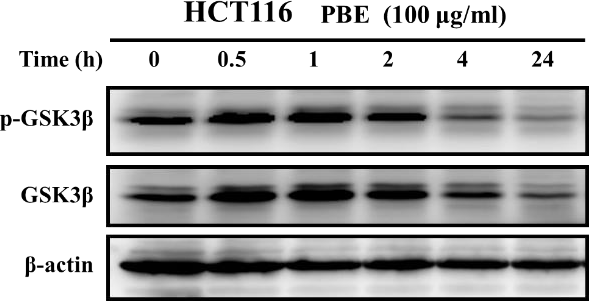


**F.7(a)**


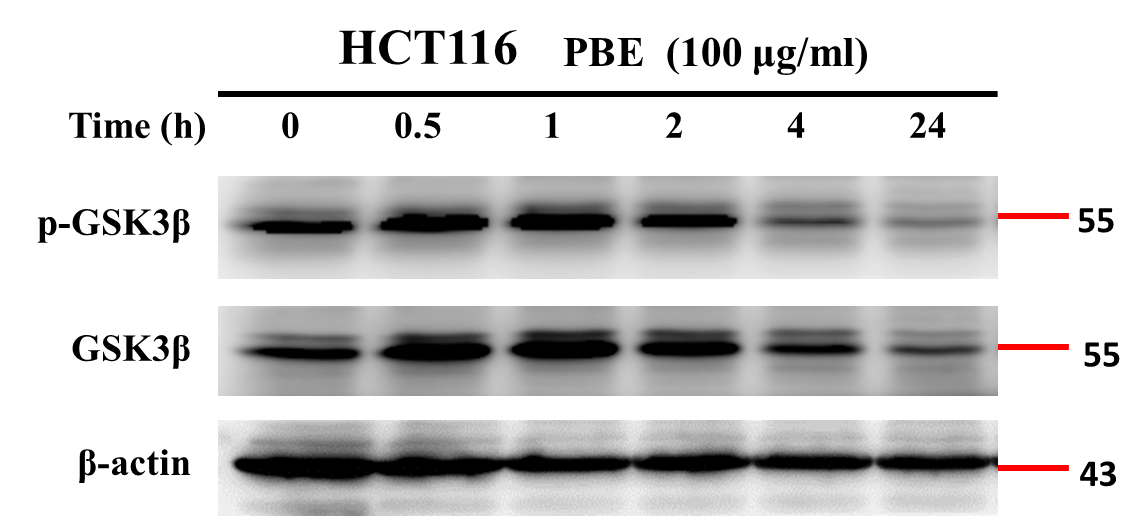


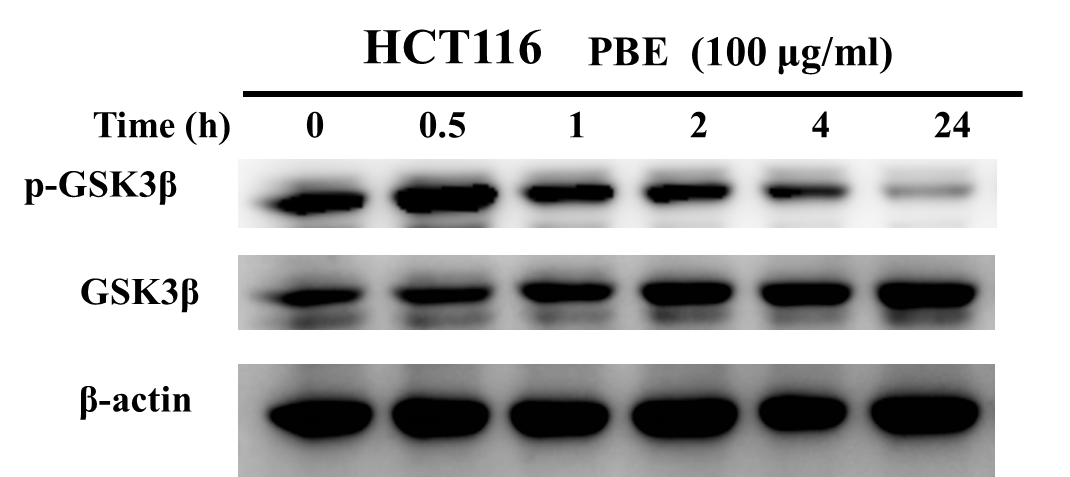


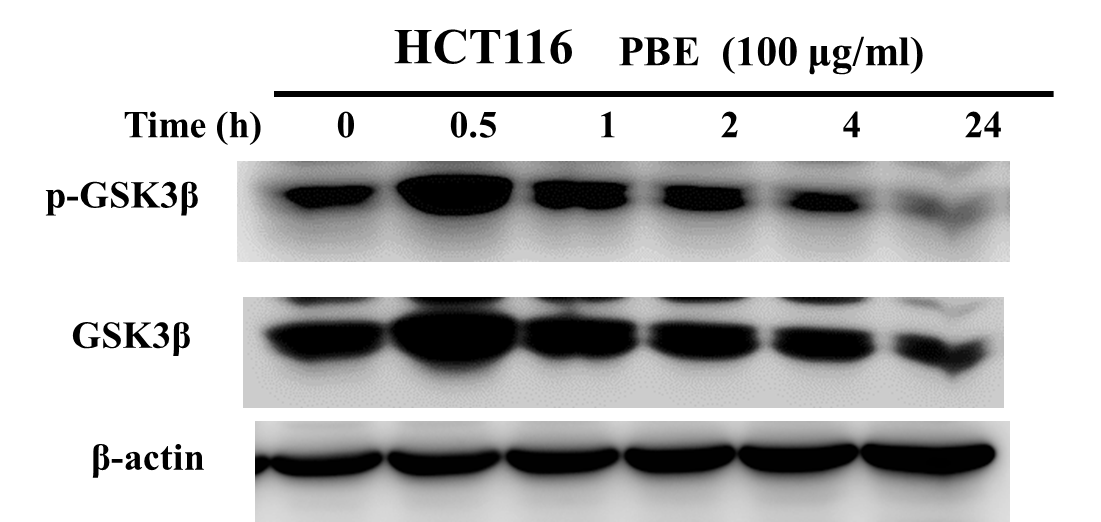


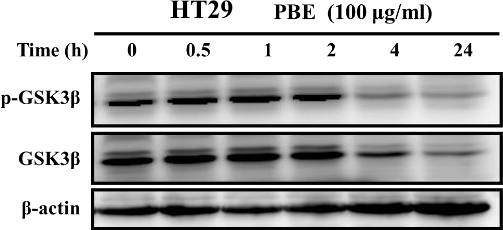


**F.7(c)**


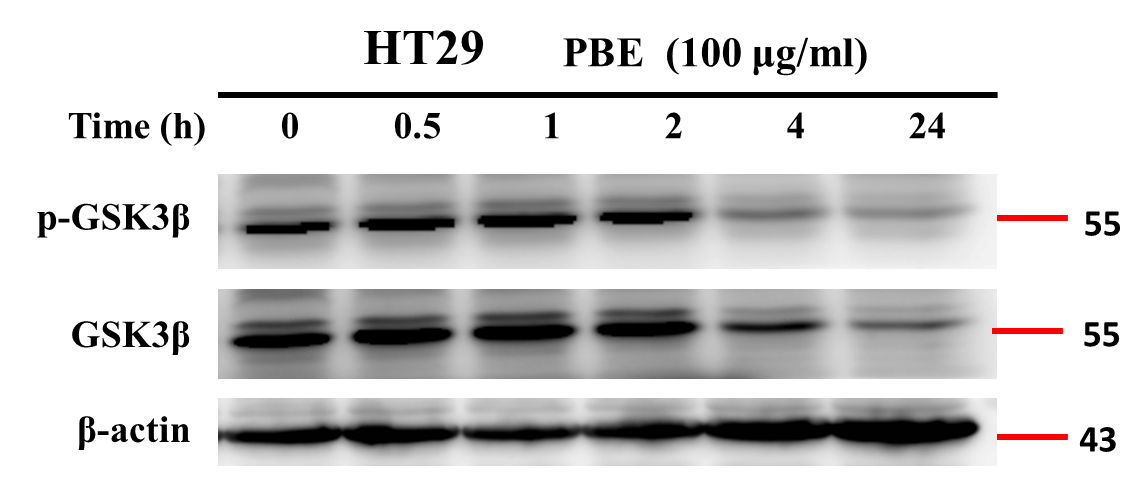


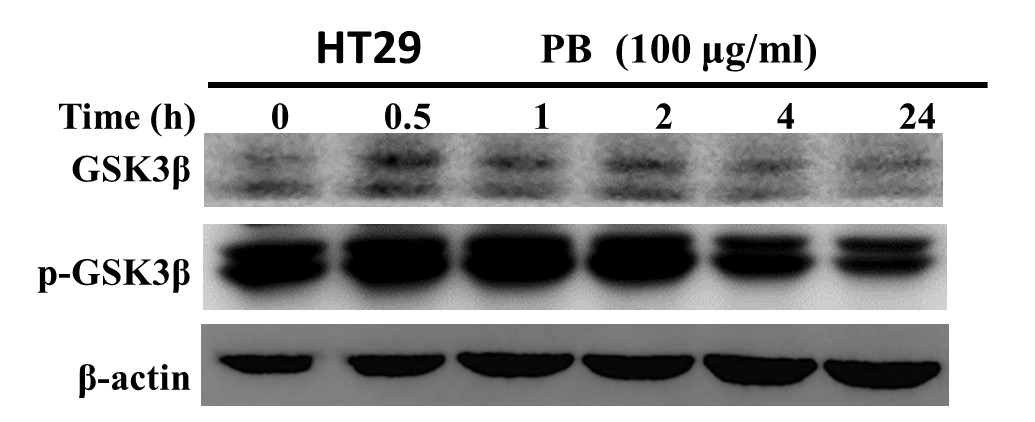


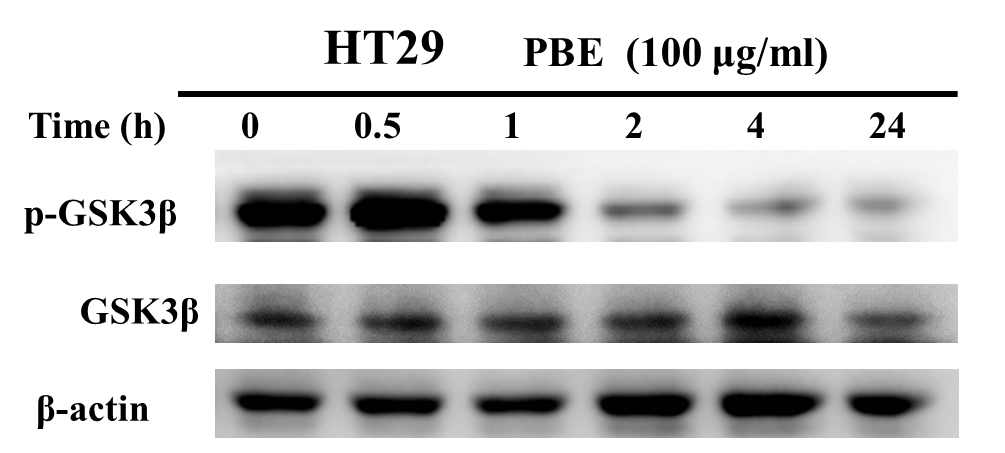


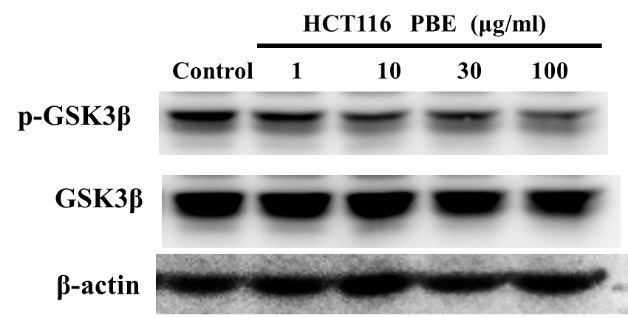


**F.7(e)**


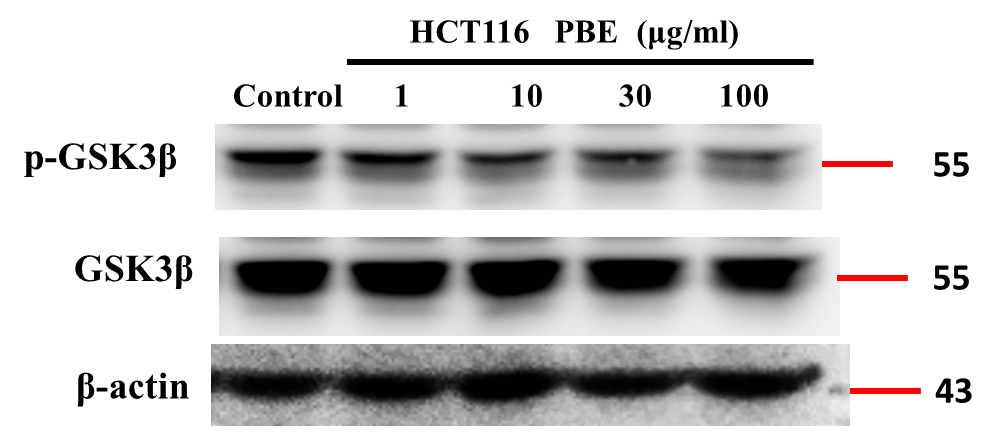


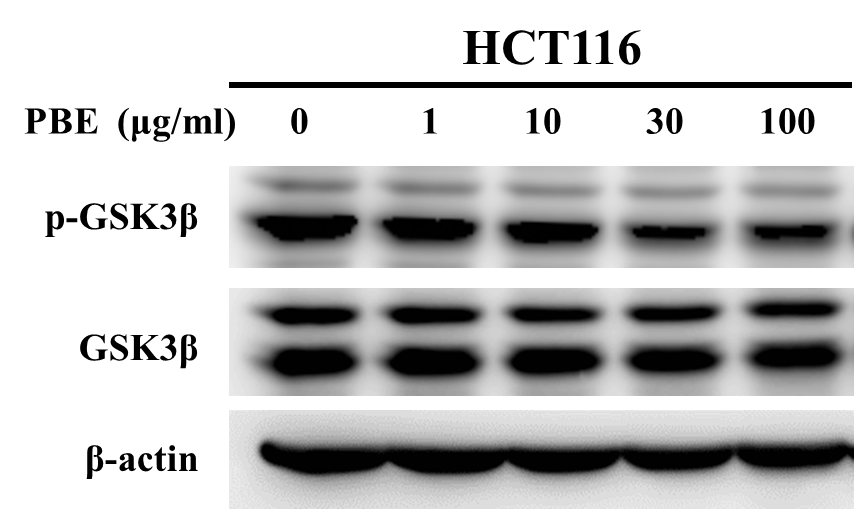


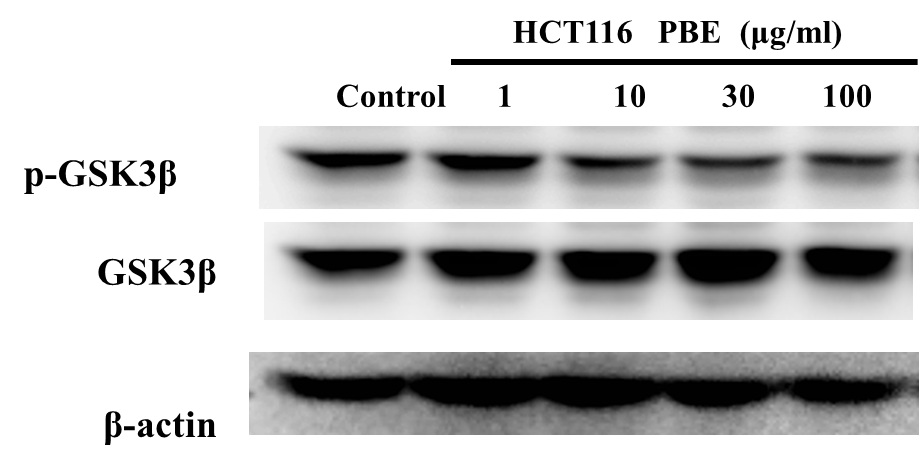


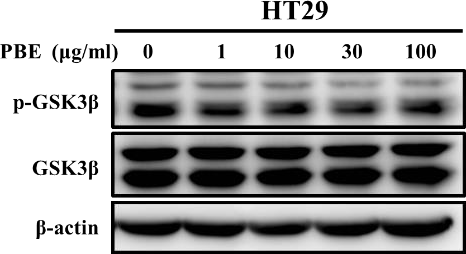


**F.7(g)**


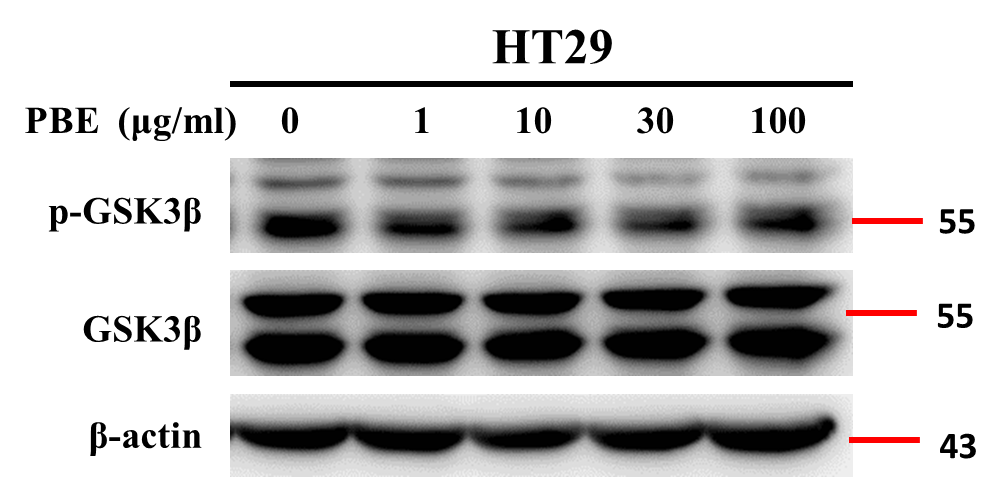

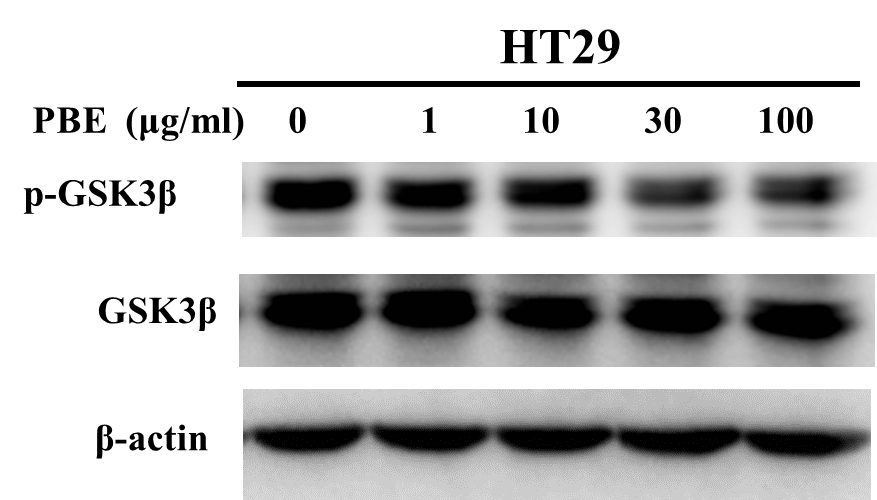


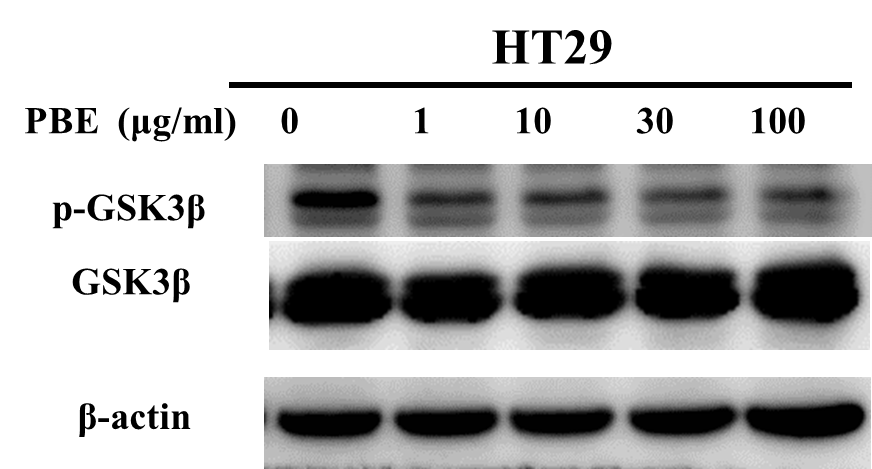


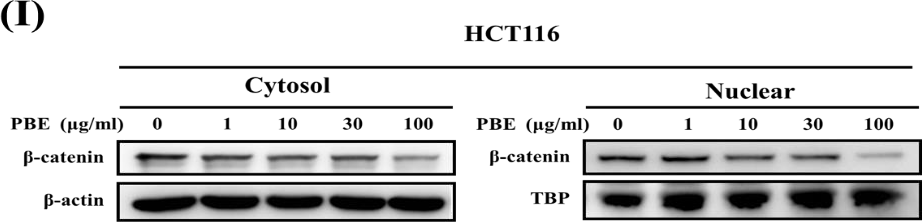


**F.7(i)**


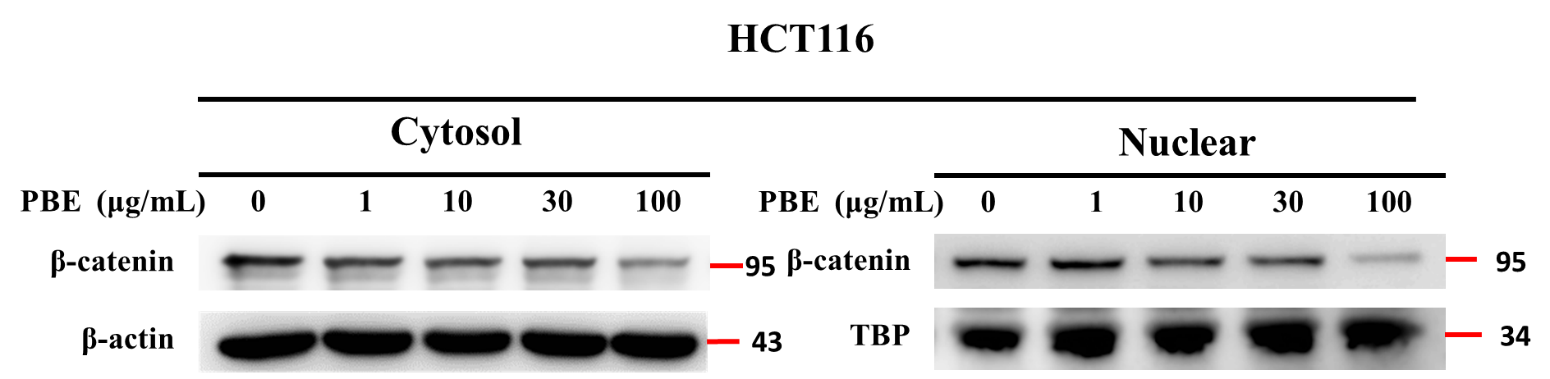


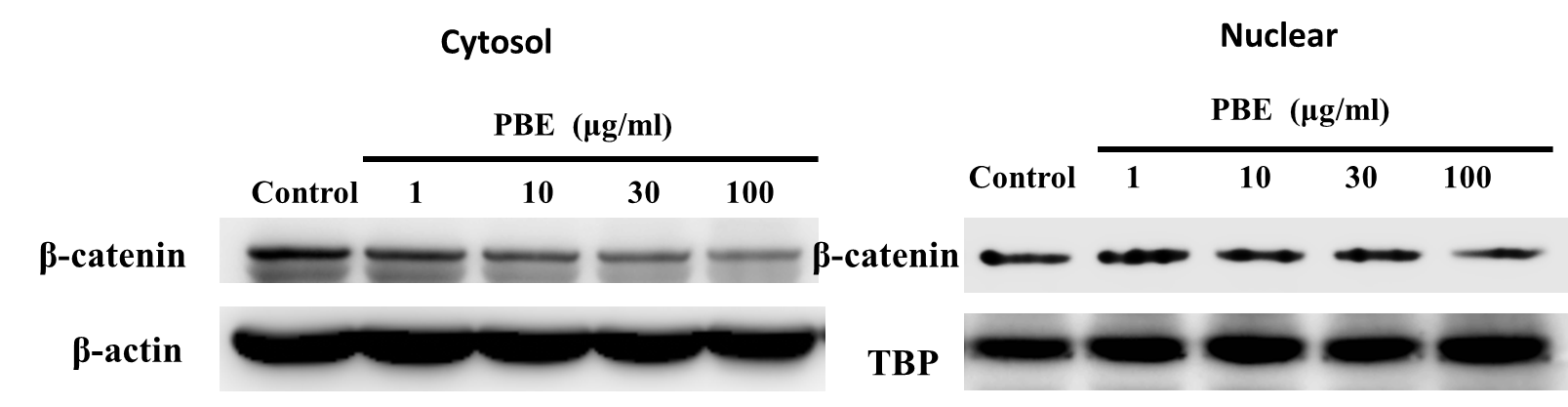


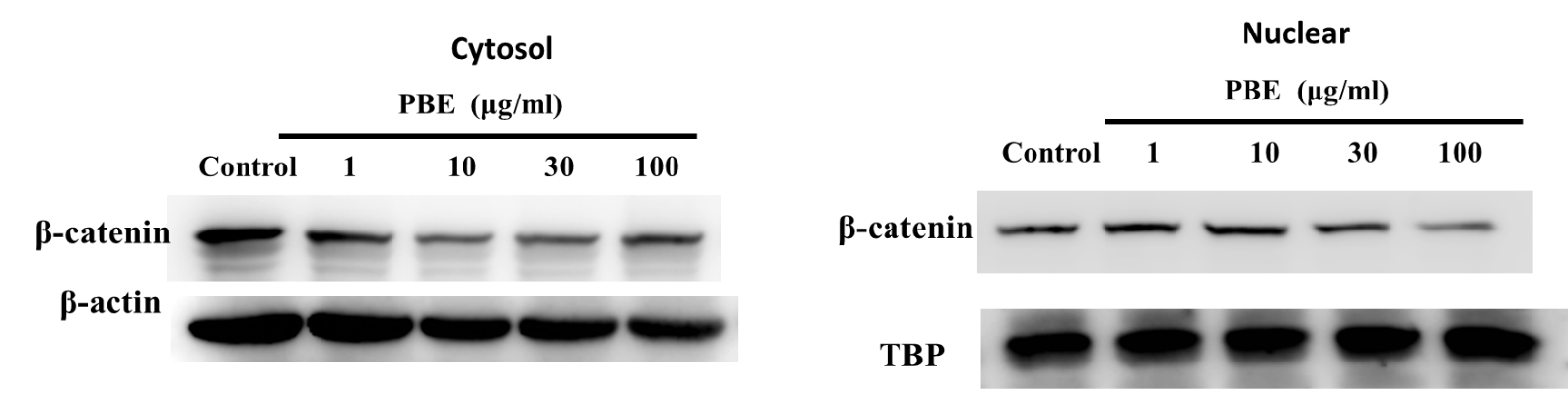


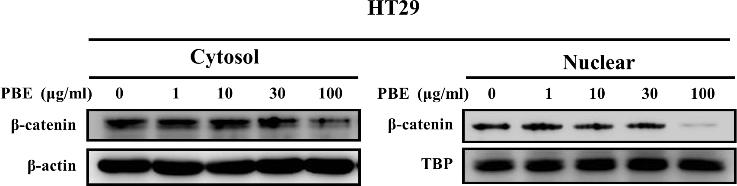


**F.7(l)**


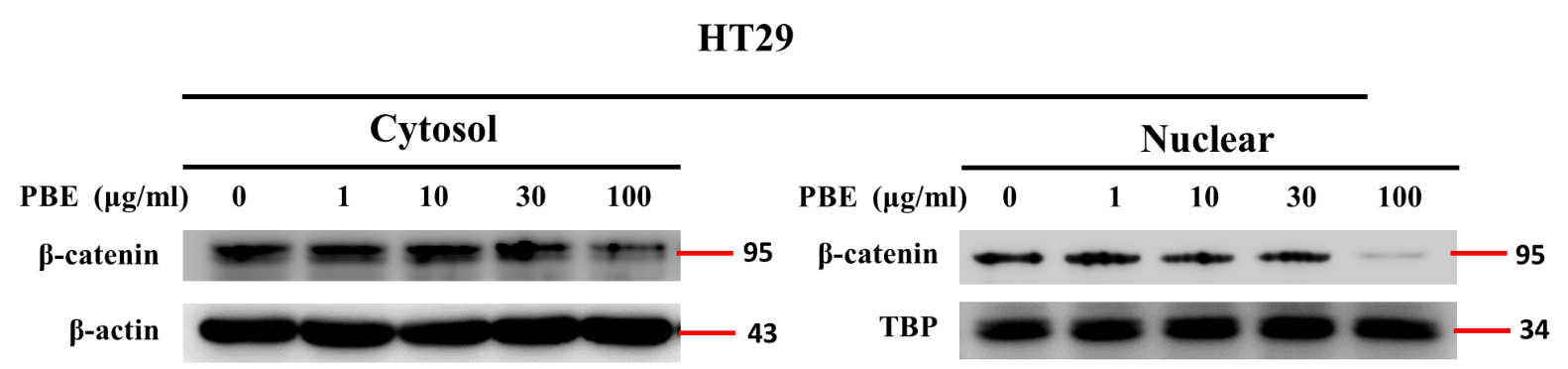


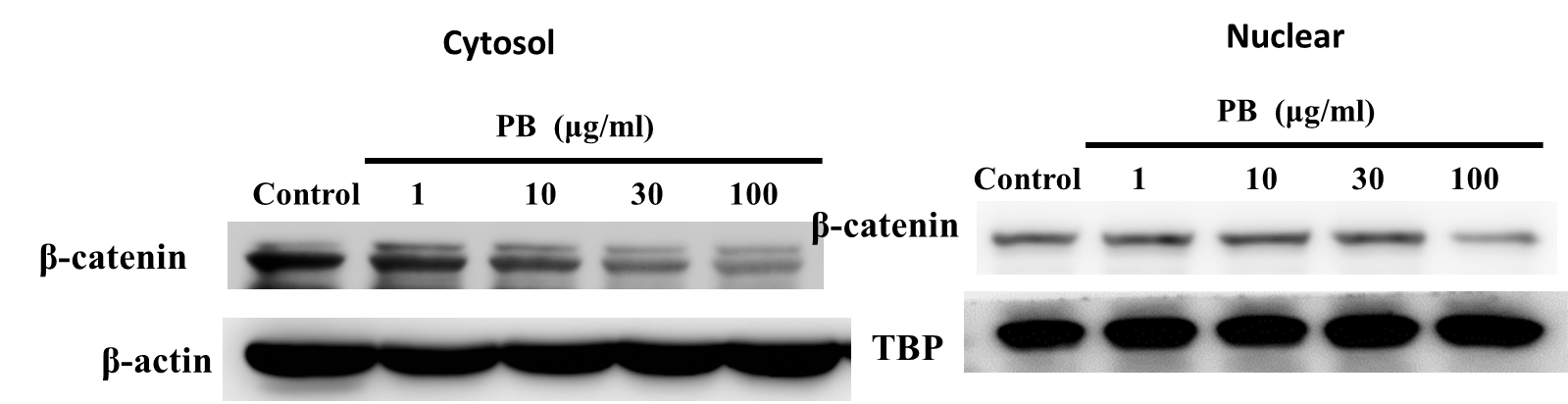


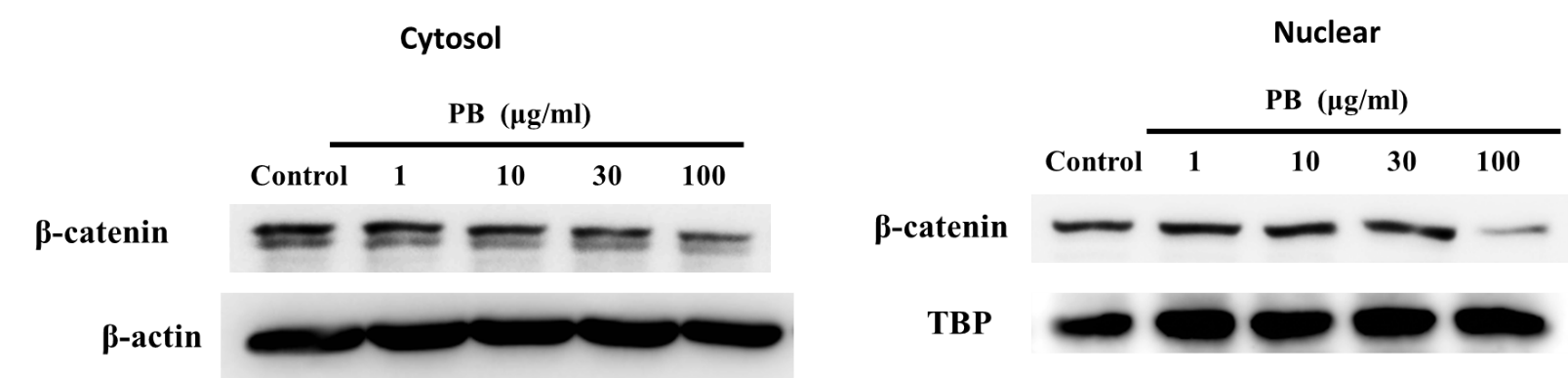


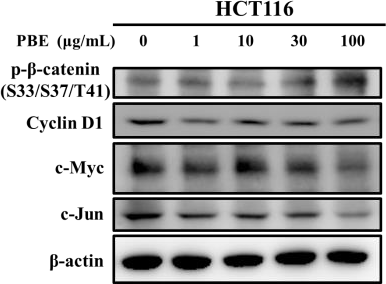


**F.7(o)**


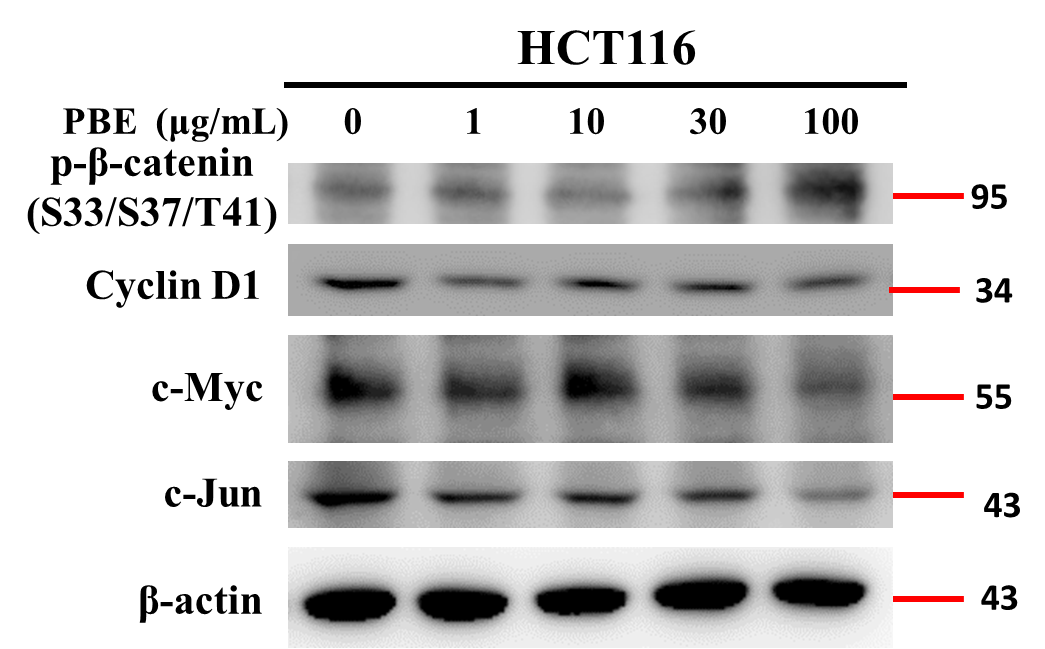


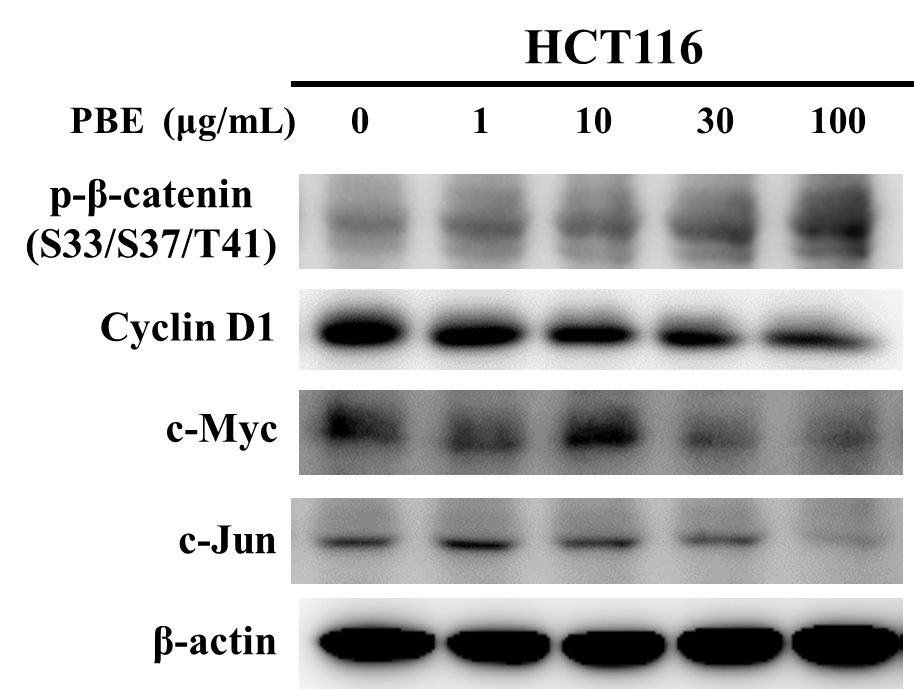


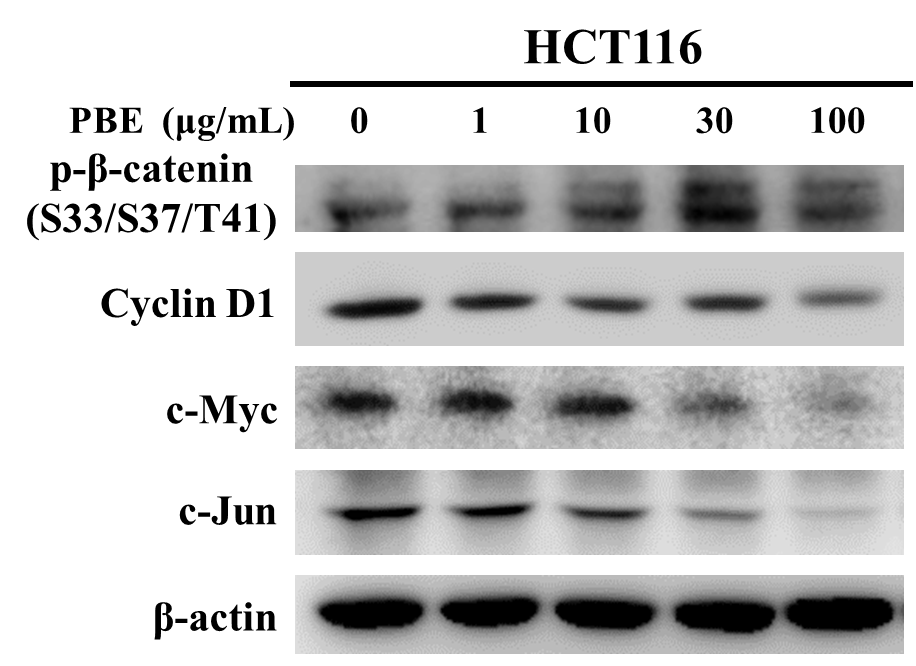


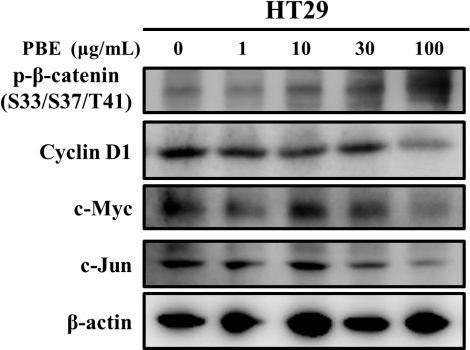


**F.7(t)**


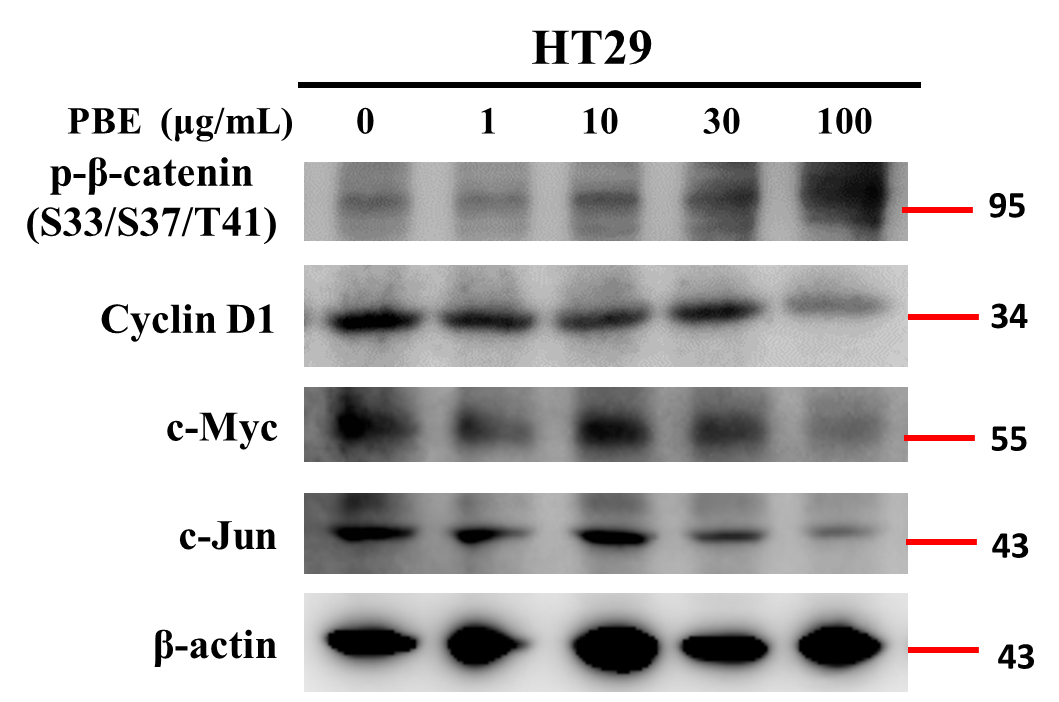


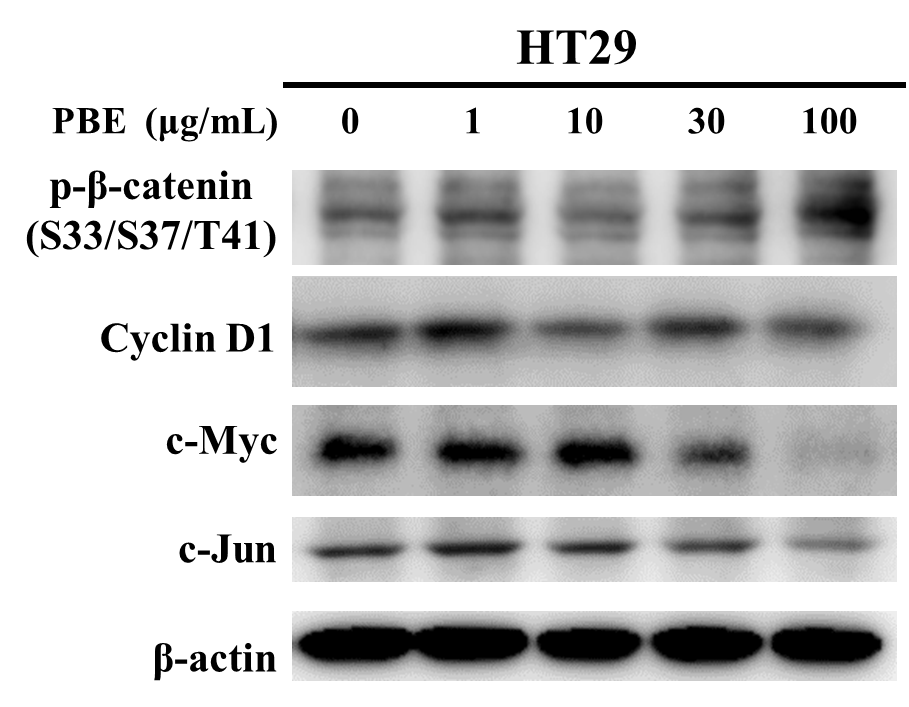


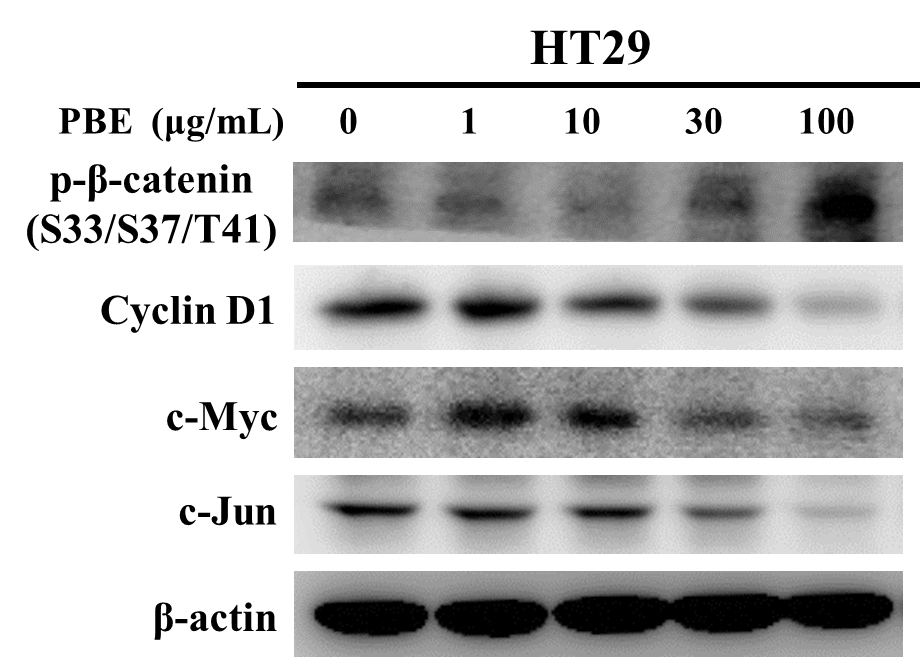

Supplement: Supplementary file 1 — Supplementary Information. [file 41598_2023_45630_MOESM1_ESM.docx]
